# Synthesis and Structure Elucidation of Two Essential Metal Complexes: In-Vitro Studies of Their BSA/HSA-Binding Properties, Docking Simulations, and Anticancer Activities

**DOI:** 10.3390/molecules27061886

**Published:** 2022-03-14

**Authors:** Jun-Li Guo, Guang-Yu Liu, Rui-Ying Wang, Shu-Xiang Sun

**Affiliations:** 1 School of Chemical Engineering, Henan Technical Institute, Zhengzhou 450042, China; gjl0192@hati.edu.cn (J.-L.G.); wry0294@hati.edu.cn (R.-Y.W.); 2 School of Chemistry and Chemical Engineering, Henan University of Technology, Zhengzhou 450001, China; gyliu@haut.edu.cn

**Keywords:** essential metal complexes, molecular structure, BSA binding, HSA binding, docking simulation, cytotoxic activity

## Abstract

Imidazole and tetrazole derivatives are widely used as clinical drugs since they possess a variety of pharmaceutical function. Zinc and iron are essential trace elements of the human body, with less toxicity and good biocompatibility. In this paper, two new essential metal mononuclear complexes [M(H_2_tmidc)_2_(H_2_O)_2_]·2H_2_O (M = Zn (**1**), Fe (**2**)) were synthesized through the reaction of 2-((1*H*-tetrazol-1-yl)methylene)-1*H*-imidazole-4,5-dicarboxylic acid (H_3_tmidc) and ZnSO_4_·7H_2_O or FeSO_4_·7H_2_O. The crystal structures were determined by means of the X-ray single crystal diffraction technique. Results from fluorescence investigations show that both complexes could interact with BSA as well as HSA through the static quenching mechanism. van der Waals forces and hydrogen bonds play important roles in the interaction of complexes and BSA/HSA since both Δ*H* and Δ*S* values are negative. The results of molecular docking are consistent with those in experimental studies. Furthermore, the anticancer activity of H_3_tmidc and both complexes against Eca-109 were preliminarily evaluated and the results show that both complexes have better anticancer activity than the corresponding ligand H_3_tmidc.

## 1. Introduction

Serum albumin (SAs) are abundant in human plasma and are often used as model proteins for studying protein–drug interactions since they can convey many endogenous and exogenous drugs [1,2,3]. The interaction of SA with potentially bioactive compounds can affect the distribution, storage, transport, pharmacodynamics, and metabolism of drugs in living organism. Protein is also regarded as one of the targets of drug therapy. Binding of serum protein with drug can be used to understand the targeted delivery and targeted release of drug molecules. Therefore, supervising the interaction of SA with latent bioactive compounds is not only of importance for drug screening in vitro, but it also affords a valuable theoretical direction for the synthesis of drug molecules [4,5,6]. In recent decades, a new interest in coordination compounds (CPs) therapy has emerged since CPs usually have better biological activity and pharmacological properties than the corresponding ligands [7,8,9,10]. However, the design and preparation of CPs are affected by several factors, and the most crucial is the choice of organic ligands and metal centers.

Tetrazole and imidazole derivatives have been widely used as clinical drugs since they possess a variety of pharmaceutical functions. For example, tetrazole compounds, such as losartan potassium, valsartan, and irbesartan, are effective and safe drugs in the treatment of primary hypertension; cefoperazone sodium can be used to treat the infective diseases of respiratory and urinogenital systems; cefazolin sodium and cefmetazole sodium have many advantages, such as broad antimicrobial spectrum, strong and fast antimicrobial effects, and little nephrotoxicity. Imidazole compounds, such as lansoprazole, (*R*)-omeprazole, pantoprazole are suitable for the treatment of gastric ulcer, duodenal ulcer, reflux esophagitis; miconazole is a highly effective and safe broad-spectrum antifungal agent, which has effects on almost all pathogenic fungi. In addition, researchers have synthesized many imidazole and tetrazole derivatives, as well as their corresponding complexes, and investigated their structures, BSA/HAS binding abilities, and antitumor activities [11,12,13,14,15,16,17,18,19,20,21,22,23,24]. For example, Cu(II) complexes [Cu(phen)(L)_2_] and [Cu(dmphen)(L)_2_] (HL = 5-benzyl-tetrazole, phen = 1,10-phenanthroline, dmphen = 4,7-dimethyl-1,10-phenanthroline) demonstrated the highest cytotoxicity against Hep2, HepG2, and MCF-7 [13]. Pd(II) complex [Pd(valp)_2_(imidazole)_2_] (valp = sodium valproate) has binding propensity to BSA and exhibits cytotoxic activity against HeLa, Hep-G2, KB, and AGZY-83a [21]. Co(II) complex [Co(Htmidc)(H_2_O)_2_]_n_ based on 2-((1*H*-tetrazol-1-yl)methylene)-1*H*-imidazole-4,5-dicarboxylic acid (H_3_tmidc) has good ability in binding with BSA [22]. H_3_tmidc contains tetrazole and imidazole groups at the same time and can offer not only *N*-donors, but also *O*-donors, in the preparation of the complexes. Moreover, the -NH group in the imidazole ring has hydrogen bond donor ability and N atoms on the imidazole/tetrazole ring possess hydrogen bond acceptability. The -CH_2_ group existing between the imidazole and tetrazole rings made H_3_tmidc more flexible and adaptive. Based on the above consideration, we selected and synthesized H_3_tmidc (see Supplementary Materials) as the ligand in this paper.

Zinc and iron are essential trace elements of the human body, with less toxicity and good biocompatibility, which are closely related to life and health. Zinc is the active center of many metal enzymes. Zinc enzymes are involved in the synthesis and decomposition of carbohydrates, lipids, proteins, and nucleic acids; they are relevant to the normal development of the body and are known as the “spark of life” [25]. Iron is an important component of hemoglobin and myoglobin is a cofactor in many enzyme reactions. Heme in hemoglobin is a complex formed by globin and Fe(II). This complex can be replaced with oxygen to form oxyhemoglobin and maintain normal physiological functions in organisms [25]. In addition, studies on the biological activities of essential metal complexes, such as antimicrobial, antiphlogistic, oxidation resisting, antidiabetic, and antineoplastic, have aroused the interest of scientific researchers [26,27,28,29,30,31].

**Scheme 1:**
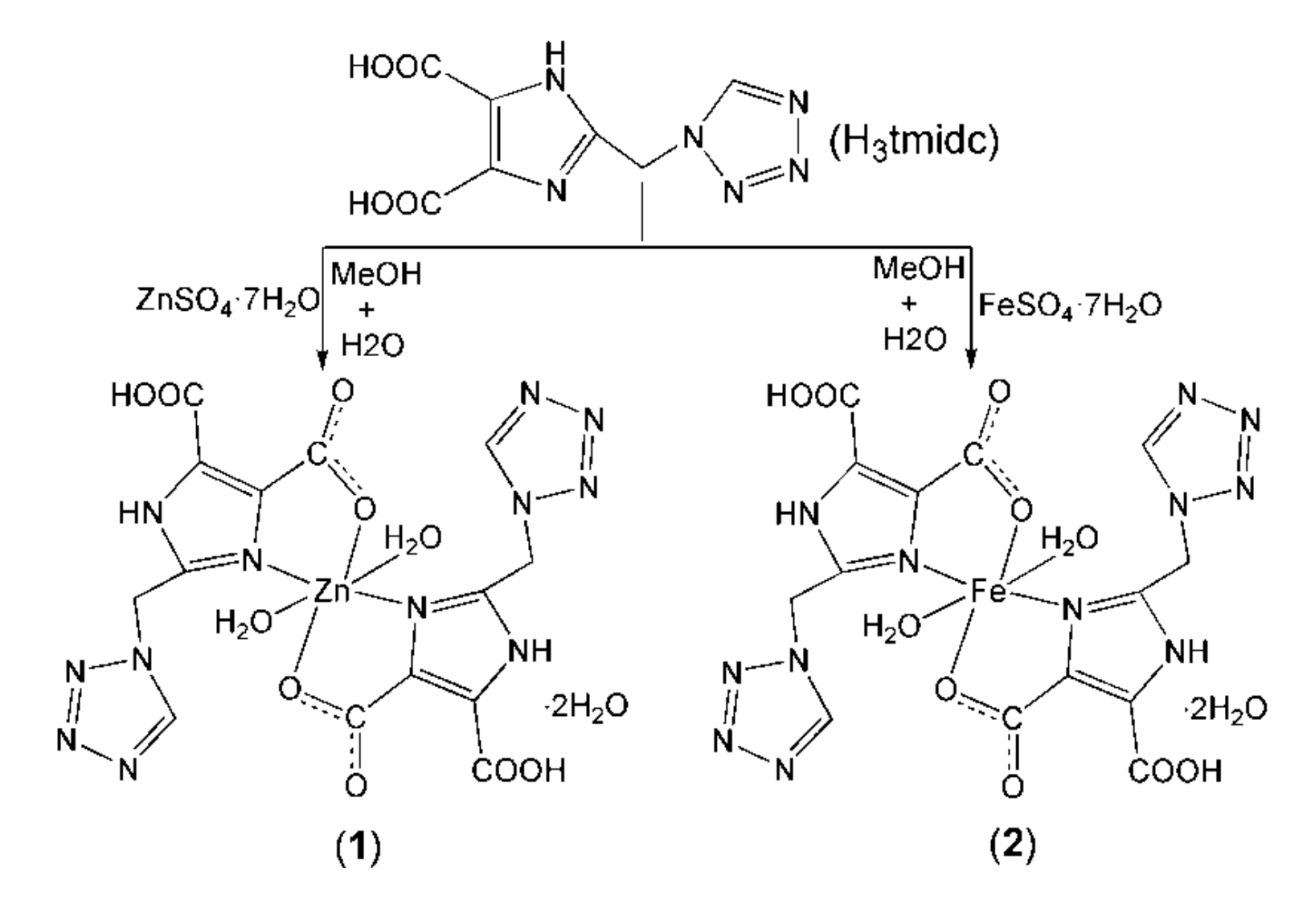
The synthetic routes of complexes **1** and **2**.

In view of the above, Zn/Fe complexes have been made through the self-assembly of H_3_tmidc with ZnSO_4_·7H_2_O or FeSO_4_·7H_2_O (Scheme 1). The complex structures were determined by a single-crystal diffractometer. The interactions between BSA/BSA and the complexes were investigated by means of the fluorescence technique. The cytotoxicity of H_3_tmidc and both complexes against Eca-109 cells were also investigated.

## 2. Results and Discussion

### 2.1. Structural Description of the Complexes

**Figure 1:**
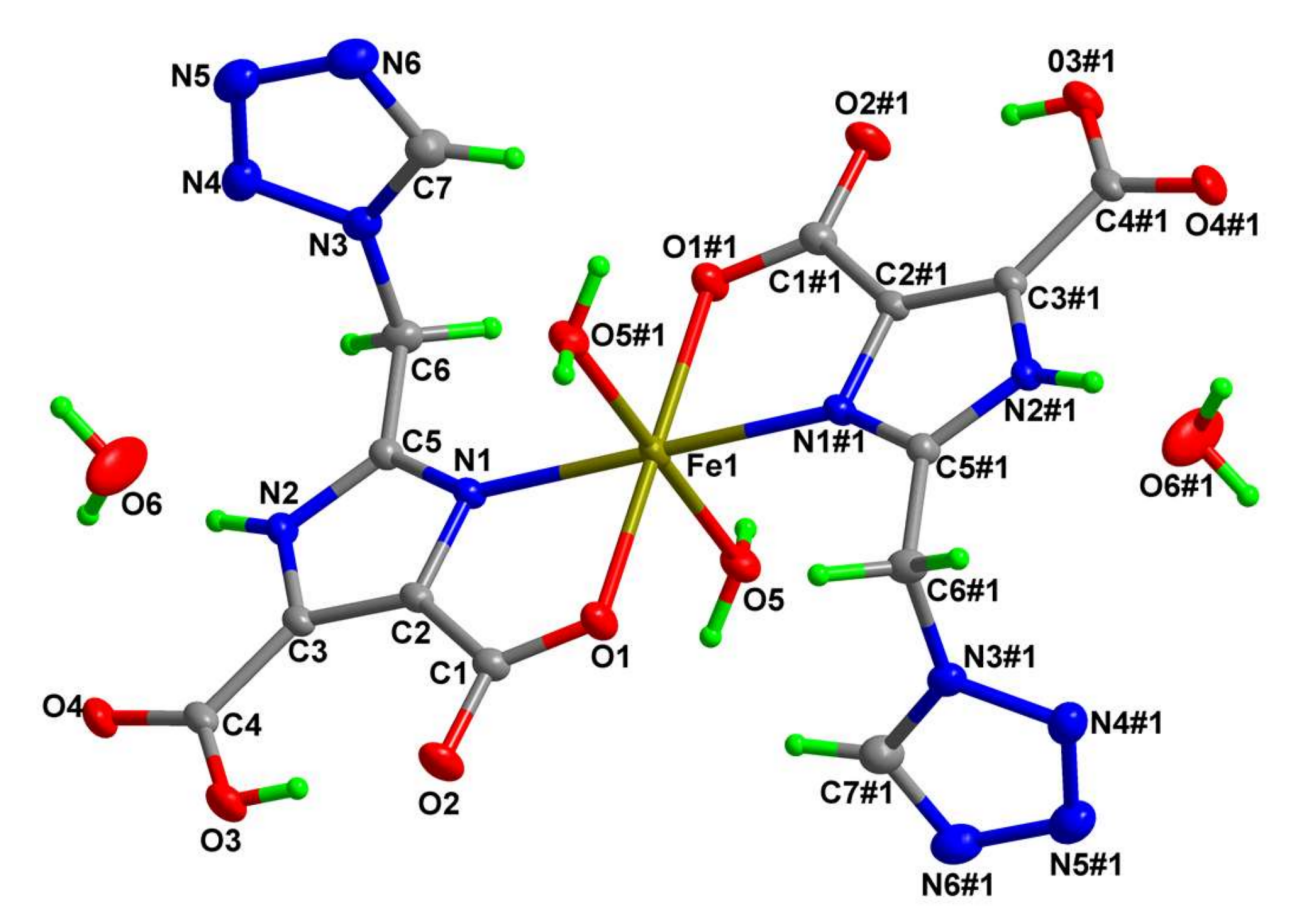
Coordination environment of the Fe(II) ion in complex **2** with the atom numbering scheme, displacement ellipsoids are drawn at the 30% probability level.

As can be seen from Table 1 and Table 2, the structures of complexes **1** and **2** are very similar, and they all crystallize in the monoclinic *P*2_1_/*C* space group. The corresponding bond lengths and bond angles in **1** and **2** are close to each other and they are isomorphous. Thus, only the structure of complex **2** is detailed here. There is one solvent water molecule, one coordinated water molecule, one H_2_tmidc^−^ anion, and one half of Fe(II) ion in each asymmetric unit. Each Fe1 is six-coordinated by N1, N1#1, O1, and O1#1 from two H_2_tmidc^−^ anions together with two H_2_O molecules (O5 and O5#1), exhibiting a slightly deformed FeN_2_O_4_ octahedral geometry (Figure 1). The imidazole ring and the chelate ring (Fe1/O1/C1/C2/N1) are almost coplanar [dihedral angle = 1.53(7)°)]. The bond angles of N1-Fe1-N1#1, O1-Fe1-O1#1, and O5-Fe1-O5#1 are 180°. The other bond angles around the Fe1 are more or less deviated from the normal values (77.39(4)–102.61(4)°). The bond length of Fe-N is 2.1407(11) Å and the bond lengths of Fe-O are 2.1031(11) and 2.1840(10) Å, respectively. It is known from the literature that these bond lengths are similar to those reported in other Fe(II) complexes, e.g., [Fe(H_2_ImDC)_2_(H_2_O)_2_]·(bpe) (H_3_ImDC is 1*H*-imidazole-4,5-dicarboxylic acid, bpe is 1,2-bi(pyridin-4-yl)ethene), with Fe–N = 2.141(6)Å and Fe–O = 2.138(5)–2.196(5) Å [32].

**Figure 2:**
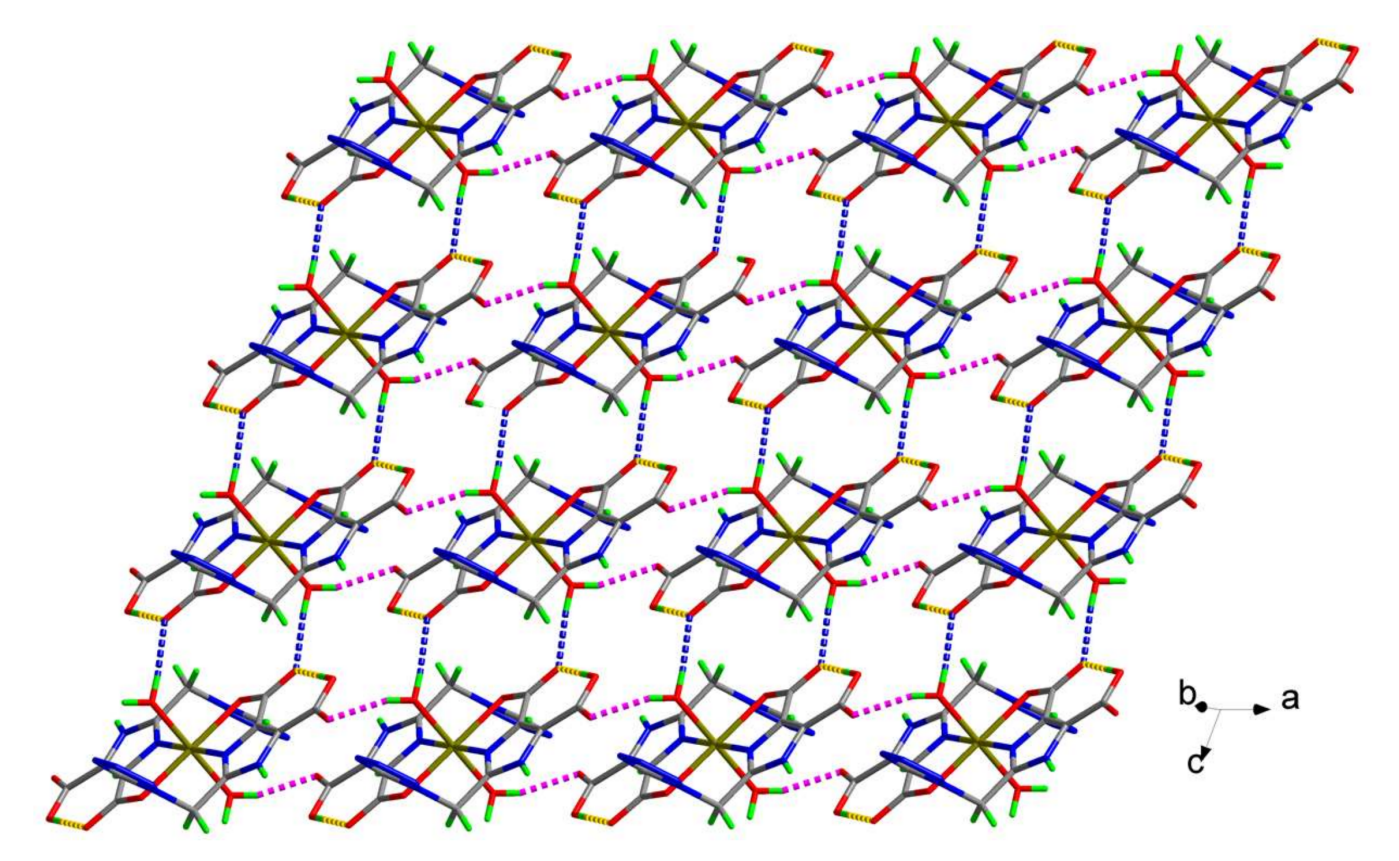
The two-dimensional structure of complex **2** linked by hydrogen bonds; yellow dashed lines represent intra-molecular hydrogen bonds, pink dashed lines represent hydrogen bonds of adjacent molecules, and blue dashed lines represent hydrogen bonds of adjacent chains.

There are five potential *N*-donors and four potential *O*-donors (two carboxylate groups) in each H_2_tmidc^−^ anion, but only one of the carboxylate groups and one of the nitrogen atoms from the imidazole ring coordinate to the central Fe(II) ion in *O*,*N*-chelating mode; while another carboxylate group, which is not deprotonated, and the other four nitrogen atoms, remain uncoordinated. In complex **2**, the imidazole ring and the tetrazole ring are not coplanar (dihedral angle = 73.46(9)°). However, the imidazole ring is nearly coplanar with the coordinated and uncoordinated carboxylate groups, the dihedral angles are 2.3(3) and 1.6(2)°, respectively. In the process of crystallization, the H_2_tmidc^−^ group has the possibility of forming hydrogen bonds since it contains both hydrogen bond donors and acceptors. Intra-molecular O3–H3⋯O2 hydrogen bonds (yellow dashed lines in Figure 2) between carboxyl and carboxylate groups stabilize the molecular configuration. An inter-molecular O5–H5A⋯O4 hydrogen bond between the coordinated H_2_O and carboxylate groups, with a bond length of 2.7757(16) Å and a bond angle of 162.0° (pink dashed lines in Figure 2), can lead to a 1D chain along the *a* direction. These 1D chains are linked by O5–H5B⋯O2 hydrogen bonds between coordinated H_2_O and carboxylate groups (blue dashed lines in Figure 2, the bond length is 2.8060(15) Å and the bond angle is 168.2°), forming a 2D layered structure. The molecular sheets are further connected through N2–H2A⋯O6, O6–H6A⋯N6, and O6–H6B⋯O4 inter-molecular hydrogen bonds (Table 3) involving carboxylate groups, imidazole rings, tetrazole rings, and solvent H_2_O, giving rise to a 3D structure in the solid state (see Figure S1 in Supplementary Materials).

The structure of complex **1** is shown in Supplementary Materials (Figures S2 and S3). As shown in Table 2, the Zn–O and Zn–N bond lengths are approximate to the values reported in the literature [33,34]. The dihedral angle between the tetrazole ring and the imidazole ring is 73.90(10)°, which is close to that in complex **2**. The dihedral angles between the planes defined by deprotonated and unprotonated carboxyl groups and the plane defined by the imidazole ring are 1.9(3) and 2.1(3)°, respectively. [Zn(H_2_tmidc)_2_(H_2_O)_2_] units are linked by hydrogen bonds among the imidazole and tetrazole rings, carboxylate and carboxyl groups, solvent and coordinated H_2_O, resulting in a 3D architecture.

Furthermore, relatively strong π–π interactions can be found between the tetrazole rings of adjacent layers. In complex **2**, the distance between two adjacent centroids is 3.4909(10) Å, which is within the normal range for π–π interactions [35,36,37]. Similar to complex **2**, the centroid–centroid distance is 3.4977(12) Å in complex **1**. Although the π–π interactions between adjacent layers are relatively weaker than the normal coordination bonds, they are essential to the crystallization of the complexes.

**Scheme 2:**
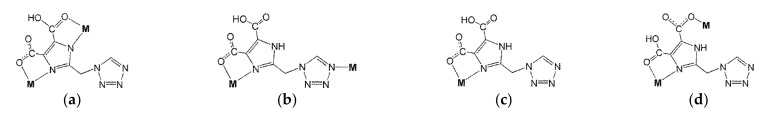
Coordination modes found in the complexes based on the H_3_tmidc ligand.

Investigating the literature, three H_3_tmidc-based complexes [Co(Htmidc)(H_2_O)_2_]_n_ (**3**), [Cd_2_(H_2_tmidc)_4_(H_2_O)_2_]·6H_2_O (**4**) and {[Ba(H_2_tmidc)_2_(H_2_O)_3_] 4H_2_O}_n_ (**5**) were reported [22,23,24]. Further study showed that their structures and the coordination modes of the H_3_tmidc-derived anions are different from complexes **1** and **2**. Complex **3** exhibits a one-dimensional structure in which Htmidc^2−^ anions coordinate to Co(II) cations in mode a (Scheme 2). Complex **4** displays a binuclear structure in which H_2_tmidc^−^ anions coordinate to Cd(II) cations in modes b and c (Scheme 2). Complex **5** shows a one-dimensional structure in which H_2_tmidc^−^ anions coordinate to Ba(II) cations in mode d (Scheme 2). In this work, by modifying the metal ions, two new mononuclear complexes [M(H_2_tmidc)_2_(H_2_O)_2_]·2H_2_O (M = Zn (**1**), Fe (**2**)) were obtained, in which H_2_tmidc^−^ anions coordinate to Zn(II) or Fe(II) cations in mode c (Scheme 2). These results indicate that H_3_tmidc is a powerful ligand, and that modifying the metal ions can influence the coordination modes of H_3_tmidc-derived anions and, thus, influence the structures and properties of the final complexes.

### 2.2. Interaction between Complexes and Albumins

#### 2.2.1. Interaction between Complexes and BSA

**Figure 3:**
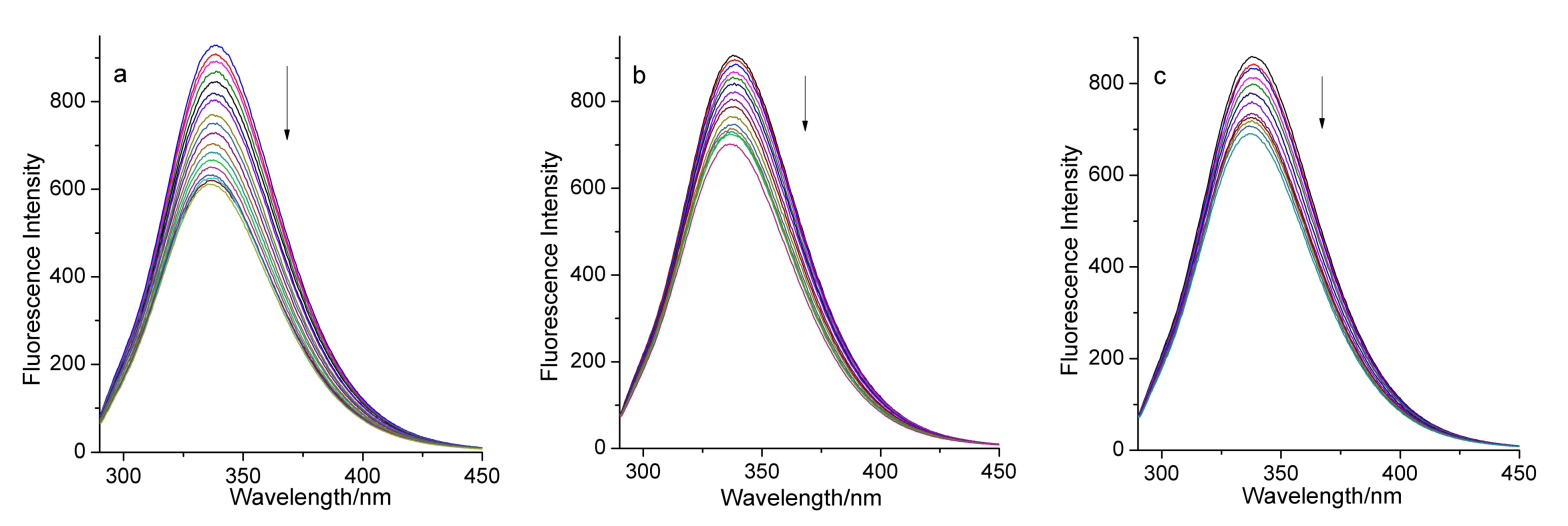
The quenching effect of complex **1** on BSA fluorescence at 298 K (**a**), 308 K (**b**), and 313 K (**c**). The arrows show the emission intensity changes upon the increasing concentrations of complex **1**.

**Figure 4:**
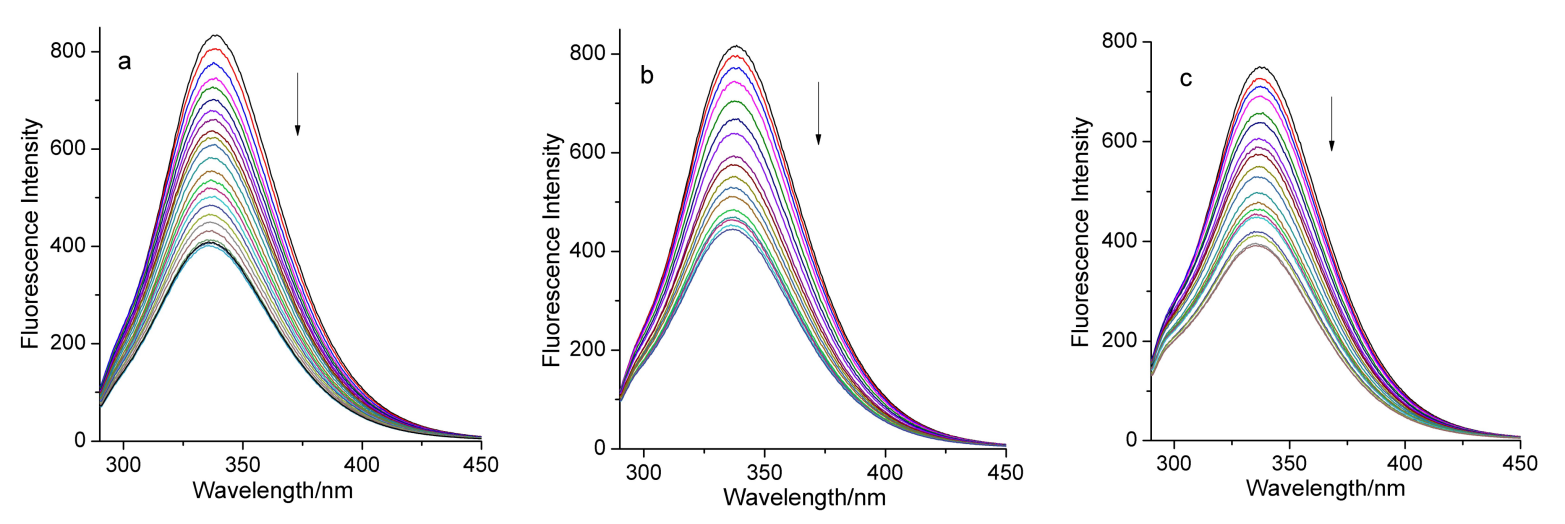
The quenching effect of complex **2** on BSA fluorescence at 298 K (**a**), 308 K (**b**), and 313 K (**c**). The arrows show the emission intensity changes upon the increasing concentration of complex **2**.

In order to investigate whether there was an interaction between complex **1** (or **2** or ligand H_3_tmidc) and BSA, we first surveyed the fluorescence spectra of BSA in the absence and presence of these compounds (Figure S4). The results show that the pure buffer solution of BSA exhibits strong fluorescence at 338 nm when excited at 280 nm. When C_BSA_:C_complex **1**_ = 1:9 or C_BSA_:C_complex **2**_ = 1:9, the fluorescence intensity of BSA decreased greatly, but the intensity declined very little when C_BSA_:C_H3tmidc_ = 1:10. This indicates that ligand H_3_tmidc hardly quenches the fluorescence of the BSA solution, but complexes **1** and **2** can significantly quench BSA’s fluorescence. Then, we investigated in detail the changes of BSA’s fluorescence intensity in different concentrations of complex **1** or **2** at 298, 308, and 313 K. As depicted in Figure 3, the intensity of the fluorescence peak of BSA decreased rapidly with increase of the concentration of complex **1**, up to 33.82%, 22.57%, and 20.67% ((*F*_0_ − *F*)/*F*_0_) of the intensity of the pure BSA solution at 298, 308, and 313 K, respectively. If complex **2** was added gradually to the BSA solution, the reduction in the fluorescence intensity of BSA was more remarkable, up to 51.26%, 45.53%, and 48.32% of the initial fluorescence intensity of BSA (Figure 4). These experimental phenomena illustrate that there are interactions between both complexes and BSA, and the addition of the complexes can quench BSA’s fluorescence. Complex **2** exhibits higher BSA quenching abilities than complex **1** [38].

The quenching mechanism can be confirmed by means of the Stern–Volmer Equation (1) [3]:

(*F*_0_ − *F*)/*F* = *K*_q_τ_0_[Q] = *K*_sv_[Q] (1)

Obviously:

*K*_sv_ = *K*_q_τ_0_ (2)

where [Q] is the concentration of the quenching agent (complex), *K*_sv_ is the Stern–Volmer quenching constant, τ_0_ is the average lifetime of the molecule without quencher, which equals 10^−8^ s [39], *K*_q_ is the quenching rate constant, *F* is the fluorescence intensity of BSA solution in the presence of the agent, and *F*_0_ is the fluorescence intensity of BSA solution in the absence of the agent. If the concentration of the complex is the *x*-axis and (*F*_0_ − *F*)/*F* is *y*-axis, the Stern–Volmer curve of the interaction between the complex and BSA can be obtained (Figures S5 and S6). According to the slope of regression curve, we can get the values of *K*_sv_. Apparently, *K*_q_ values can be given by Equation (2). As data listed in Table 4, the values of *K*_sv_ and *K*_q_ are inversely proportional to temperature and the *K*_q_ values are larger than the maximum dynamic *K*_q_ value (2.0 × 10^10^ L mol^−1^ s^−1^) [38]. These suggest that the quenching of BSA by complex 1 or 2 is the static quenching mechanism [40,41].

When the small molecule combines with the protein, the number of bonding sites, n, and the apparent binding constant, *K*_b_, can be obtained from Equation (3) [41].

Log[(*F_0_ − F*)/*F*] = log*K_b_* + nlog[Q] (3)

If we plot log[(*F_0_ − F*)/*F*] against log[Q] (Figures S7 and S8), the values of n and *K*_b_ can be obtained from the corresponding slope and intercept. The data in Table 4 show that the *K*_b_ values decrease as the temperature rises, which may imply that the binding ability between each complex and BSA decreased with the rising of temperature. The binding constants between complex **2** and BSA are greater than those of complex **1**, which indicate that the interaction force between complex **2** and BSA is stronger than that between complex **1** and BSA. The number of binding sites of each complex with BSA is close to 1. This illustrates that there is one binding site between each complex and BSA [41].

The main forces between drug and BSA largely include hydrogen bond, van der Waals force, electrostatic force, and hydrophobic force. According to the sign of enthalpy change (Δ*H*) and entropy change (Δ*S*), the type of force between the drug and BSA can be judged. The negative values of Δ*H* and Δ*S* imply the van der Waals forces and hydrogen bond interactions between BSA and the drug; the positive values of Δ*H* and Δ*S* suggest a hydrophobic interaction between BSA and the drug; the negative value of Δ*H* and the positive value of Δ*S* reflect an electrostatic force between BSA and the drug. If the temperature range is small, we can suppose that Δ*H* and Δ*S* are constant. Equation (4) describes a straight line with a slope of −Δ*H*/*R* and a *y*-intercept of Δ*S*/*R* [41].

ln*K*_b_ = (−Δ*H*/*RT*) + (Δ*S*/*R*) (4)

where *R* is the molar gas constant, *T* is the Kelvin temperature. Δ*G* can be calculated by means of Equation (5):

Δ*G* = Δ*H* − *T*Δ*S* (5)

The values of Δ*H*, Δ*S*, and Δ*G* between the interaction of complex **1** (or **2**) and BSA are summarized in Table 4. Since all of the values of Δ*G* are negative, the binding processes at the three temperatures are spontaneous. The change tendency of Δ*G* values suggest that the spontaneity decreases with the increase of temperature (from 298 to 313 K). The negative value of Δ*H* implies that the binding process is exothermic and is basically enthalpy-driven [41]. The negative values for both Δ*H* and Δ*S* forecast that hydrogen bonding and the van der Waals force are the main forces between the complex and BSA here.

**Figure 5:**
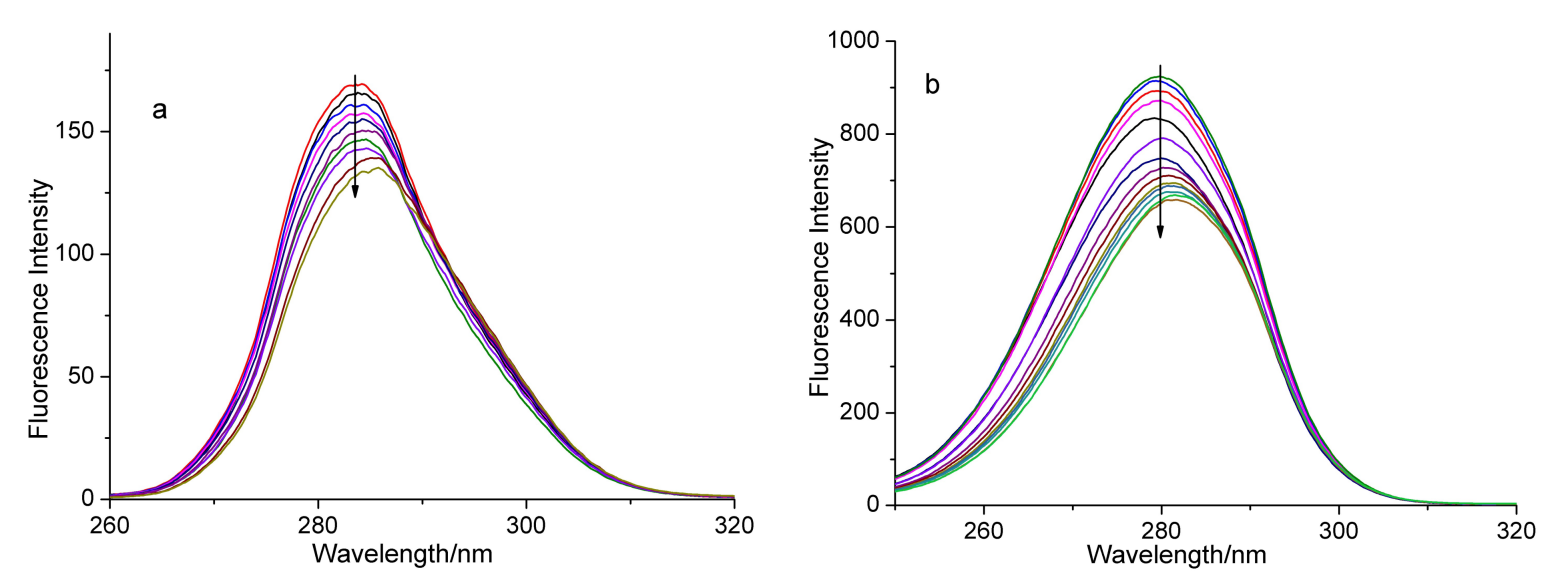
Synchronous fluorescence spectra of BSA in the presence of increasing amounts of complex **1** at Δλ = 15 nm (**a**) and at Δλ = 60 nm (**b**). The arrows show the emission intensity changes upon the increasing concentrations of complex **1**.

**Figure 6:**
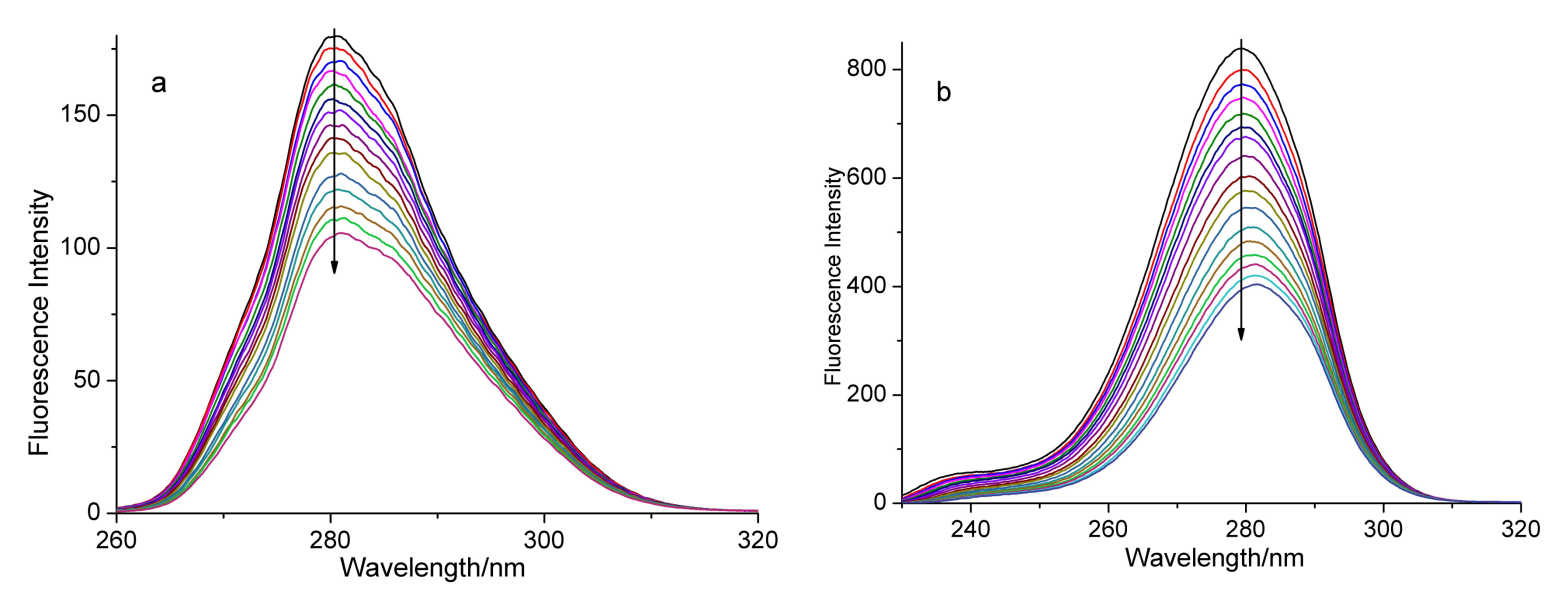
Synchronous fluorescence spectra of BSA in the presence of increasing amounts of complex **2** at Δλ = 15 nm (**a**) and at Δλ = 60 nm (**b**). The arrows show the emission intensity changes upon the increasing concentrations of complex **2**.

Synchronous fluorescence is the scanning of excitation and the emission, at the same time, and the wavelength difference (Δ*λ*) between them is fixed. Synchronous fluorescence spectrum could provide crucial information for conformational changes of the protein [42]. The luminescence of the BSA solution mainly comes from tyrosine and tryptophan. In general, the synchronous fluorescence spectrum obtained at Δ*λ* = 15 nm can express the characteristic spectrum of tyrosine, and the spectra acquired at Δ*λ* = 60 nm are the features of tryptophan residues [43]. For the sake of intensive study, the influence of the addition of the complex on the BSA conformation and the synchronous fluorescence spectra of BSA were determined. Figure 5 and Figure 6 present the synchronous fluorescence spectra of the BSA solution upon gradual addition of the test complex at Δλ = 15 nm and Δλ = 60 nm. Obviously, the fluorescence intensity of Figure 5b and Figure 6b decreased more than those of Figure 5a and Figure 6a, suggesting that the quenching degree of both complexes to tryptophan is larger than that of tyrosine. Moreover, the maximum emission wavelengths have slight red shifts with the addition of the complex. These results suggest that the interaction between complex **1** (or **2**) and BSA can affect the conformation of both tryptophan and tyrosine regions in BSA [41].

#### 2.2.2. Interaction between Complexes and HSA

Similar to BSA, a pure HSA solution exhibits a strong fluorescence emission peak at 332 nm upon excitation at 280 nm. The addition of complex **1** to the solution of HSA can also result in fluorescence quenching (Figure S9, up to 24.87%, 28.34%, and 18.32% of the initial fluorescence intensity of HSA at 298, 308, and 313 K, respectively). While the quenching induced by complex **2** to the HSA fluorescence is much more pronounced than complex **1** (Figure S10, up to 51.66%, 54.14%, and 41.64% of the initial fluorescence intensity of HSA at 298, 308, and 313 K, respectively). This indicates that the intrinsic fluorescence of the HSA can be quenched by the addition of the test complex.

The Stern–Volmer plots for the interactions between complex **1** or **2** with HSA are given in Figures S11 and S12. The relevant *K*_sv_ and *K*_q_ values are calculated from the slope of the regression curve and listed in Table 5. These results suggest that both complexes exhibit good HAS quenching abilities. All of the *K*_q_ values at the experimental temperatures are larger than 2.0 × 10^10^ L mol^−1^ s^−1^, which implies that the fluorescence quenching of HSA by both complexes belongs to static quenching. The related *K*_b_ and n values can be calculated by means of Figures S13 and S14 and are listed in Table 5. These *K*_b_ values are relatively lower than those of the BSA-complex **1** (or **2**) system. Similar to the BSA-complex **1** (or **2**) system, the n values at the three temperatures are close to 1. That is to say, there is only one binding site in HSA for complex **1** or **2**. The van’t Hoff equation can be used to estimate the thermodynamic parameters of the HSA-complex **1** (or **2**) system and the calculated results are given in Table 5. The binding process of complex **1** (or **2**) to HSA is mainly due to hydrogen bonding and van der Waals forces since Δ*H* and Δ*S* are negative. The interactions between complex **1** (or **2**) and HSA at the tested temperatures are spontaneous in thermodynamics as their Δ*G* values are negative. Synchronous fluorescence spectra of the HSA-complex **1** (or **2**) system are depicted in Figures S15 and S16. Similar to the BSA-complex **1** (or **2**) system, as the concentration of the complex increases and the fluorescence intensity of HSA solution decreases accompanied by a slight red shift in maximum emissions. These suggest that the conformations of tryptophan and tyrosine residues in HSA changed in the process of the interaction between complex **1** (or **2**) and HAS.

In the three reported H_3_tmidc-based complexes [22,23,24], researchers investigated the BSA-binding properties of complexes [Co(Htmidc)(H_2_O)_2_]_n_ (**3**) and [Cd_2_(H_2_tmidc)_4_(H_2_O)_2_]·6H_2_O (**4**). Further study showed that the Stern–Volmer quenching constants (*K*_sv_) of complex **1** are slightly smaller than those of complexes **3** and **4**, but the *K*_sv_ values of complex **2** are slightly larger than those of complexes **3** and **4** [22,23]. These indicate that complex **1** exhibits a lower BSA quenching ability than complexes **3** and **4**, while complex **2** displays higher BSA quenching ability than complexes **3** and **4**. In addition, by comparing the *K*_b_ values of complexes **1**–**3**, one can see that the binding ability of complex **2** for BSA is close to that of complex **3**, while complex **1** exhibits lower binding affinity for BSA than complex **3**. By comparing the *K*_b_ values in Table 4 and Table 5, one can notice that both complexes **1** and **2** present higher binding affinity for BSA/HAS and complex **2** possesses stronger binding ability for BSA/HSA than complex **1**. These indicated that center metal ions may affect the interaction between complexes and BSA/HSA. More complexes based on H_3_tmidc need to be synthesized in the future to discuss how the effects of metal ions on the quenching affect the fluorescence of the BSA/HAS.

### 2.3. Molecular Docking

**Figure 7:**
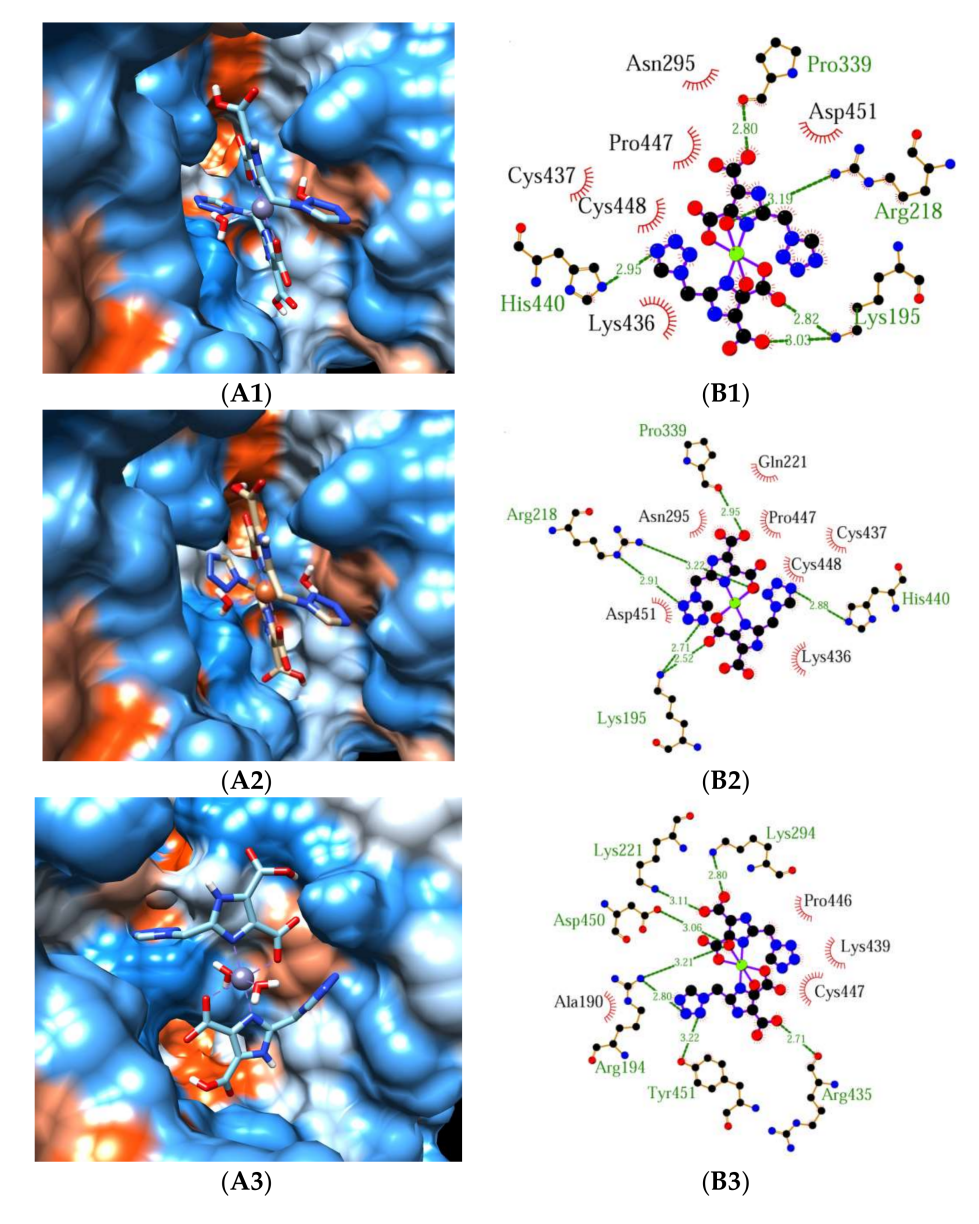

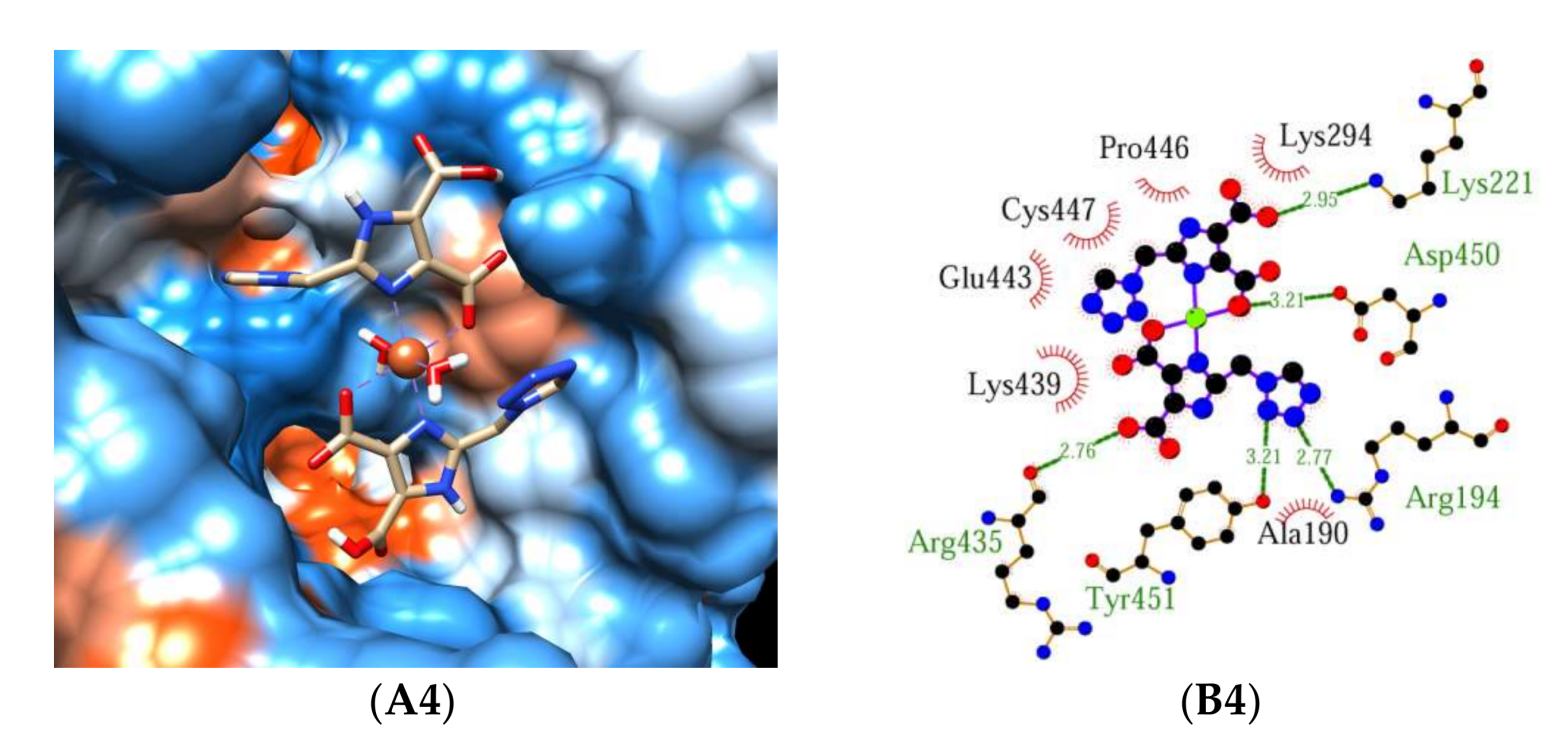
The binding mode of complex **1**-HSA (**A1**,**B1**), complex **2**-HSA (**A2**,**B2**), complex **1**-BSA (**A3**,**B3**), complex **2**-BSA (**A4**,**B4**). Complex **1** or **2** is shown as the cylinder mode and HSA/BSA is represented by the surface model (**A1**–**A4**). Hydrogen bonds between docked complex **1** or **2** and amino acid residues of HSA/BSA (**B1**–**B4**). H-bonds are shown as green lines, the residues that form the hydrogen bond with the complex are labeled in green font, and the residues related to the hydrophobic interaction are labeled in black font.

The structure of complexes and the interactions between complex and HSA/BSA predicted by molecular docking are shown in Figure 7. Figure 7A1–A4 shows the best conformation of the binding mode between complex **1** or **2** and HSA/BSA. Figure 7B1–B4 illustrates the hydrogen bonds between complex **1** or **2** and amino acid residues of HSA/BSA. As depicted in Figure 7A1,A2,B1,B2, both complexes have similar interaction modes with HSA. Five hydrogen bonds formed between complex **1** and Arg218, Pro339, His440, Lys195, and six hydrogen bonds formed between complex **2** and Arg218, Pro339, His440, Lys195. In addition, although the above thermodynamic studies show that hydrogen bonds are the main driven forces for the interaction of complex **1** or **2** with HSA, the possibility of hydrophobic forces cannot be excluded. As shown in Figure 7B1,B2, hydrophobic interactions can be observed between complex **1** and Asn295, Pro447, Cys437, Cys448, Lys436, Asp451, and between complex **2** and Gln221, Asn295, Pro447, Cys437, Cys448, Lys436, Asp451. As shown in Figure 7B3, seven hydrogen bonds formed between complex **1** and Arg194, Lys221, Asp450, Tyr451, Arg435, and Lys294. Hydrophobic interactions are mainly formed between complex **1** and Ala190, Lys439, Pro446, Cys447. Similar to complex **1**, five hydrogen bonds can be observed between complex **2** and Arg194, Lys221, Asp450, Tyr451, and Arg435. The residues contribute to the hydrophobic interactions are Ala190, Lys294, Lys439, Pro446, Cys447, and Glu443 (Figure 7B4). A comparison between Figure 7A1,A2 and Figure 7A3,A4 indicates that the active pocket in BSA is wider than that in HSA, and this can result in the stronger binding ability between complex **1** (or **2**) and BSA, which is in accordance with the smaller experimental Δ*G* and lager *K*_b_ for BSA.

### 2.4. In Vitro Anticancer Activities

Relatively strong bonding abilities between both complexes and BSA/HSA inspired us to study the anticancer abilities of the complexes [7,8]. In this paper, we primarily investigated the anticancer activities of ligand H_3_tmidc and both complexes against human esophageal carcinoma cell (Eca-109) through MTT assay. The blank control group only added RPMI-1640 complete medium and DMSO (the concentration is 0.1%). Cisplatin was selected as the positive control group and its final concentrations were 83.33, 73.33, 63.33, 53.33, 43.33, 33.33, 23.33, 13.33, and 3.333 μmol∙L^−1^, respectively. The final concentrations of H_3_tmidc and complex **1** were 100, 80, 50, 10, 5, 1, 0.5, 0.1, and 0.01 μmol∙L^−1^, respectively. The final concentrations of complex **2** were 45, 30, 15, 4.5, 0.90, 0.45, 0.045, 0.03, and 0.01 μmol∙L^−1^, respectively. The experimental results indicate that the inhibitory effects of the ligand and both complexes on Eca-109 increased with the increase of concentrations, but the inhibition rates of the tested compounds did not reach 50% due to their low solubility. Thus, the IC_50_ values for H_3_tmidc, complexes **1** and **2,** were calculated from SPSS17.0 statistical software, and they are 231, 145, and 160 μmol∙L^−1^ for H_3_tmidc, complexes **1** and **2**, respectively. Under the same conditions, the IC_50_ value of cisplatin is 57.4 μmol∙L^−1^. Compared with cisplatin, H_3_tmidc and both complexes appeared to be significantly inactive against Eca-109. Zinc sulfate and ferrous sulfate have no obvious biological activity even up to 400 μmol∙L^−1^. This implies that the observed cytotoxic properties of both complexes may come from the chelation of the ligand with the metal ions [7,44]. Since the inhibition rate is not high, we do not discuss the anticancer activity of the complexes in more detail.

## 3. Materials and Methods

### 3.1. Materials and Instrumentation

Bovine serum albumin (BSA) and human serum albumin (HSA) were purchased from Beijing’s Aoxing Biotechnology Co., Ltd. (Beijing, China), and were used without further purification. Eca-109 was presented by Professor Yanqin Zhu, School of Basic Medicine, Henan University of Chinese Medicine (Zhengzhou, China). Tris and DMSO were supplied by the Sinopsin Group Chemical Reagent Co., Ltd. (Shanghai, China). Roswell Park Memorial Institute-1640 (RPMI-1640) medium and fetal bovine serum (FBS) came from Beijing Solarbio Co., Ltd. (Beijing, China). Cisplatin was purchased from Qilu Pharmaceutical Co., Ltd. (Hainan, China). Double distilled water was used throughout the whole experiment process. The reagents involved in this experiment were analytically pure and were not purified further prior to use. Determination of C, H, and N contents in the complexes were carried out on a FLASH EA 1112 elemental analyzer (Thermo Fisher Scientific Company, Waltham, MA, USA). ^1^H NMR spectra were recorded on a Bruker Avance-400 NMR spectrometer (Billerica, MA, USA) at room temperature in DMSO. An F-7000 spectrofluorometer (Hitachi, Ltd., Chiyoda-ku, Tokyo, Japan) was used to study the bonding ability between complexes and BSA/HSA. The excitation and emission slits were both set at 5 nm. MTT assays were carried out on an iMark microplate reader (Bio-Rad Laboratories, Shanghai, China).

### 3.2. Syntheses of the Complexes

#### 3.2.1. Syntheses of Complex [Zn(H_2_tmidc)_2_(H_2_O)_2_]·2H_2_O (**1**)

H_3_tmidc (0.02 mmol) was dissolved in methanol solution (2 mL) and slowly dripped in to a 2 mL water solution that contained ZnSO_4_·7H_2_O (0.02 mmol). A clear solution was obtained after filtration and placed in a closed container at room temperature. Light yellow crystals of high quality were obtained after one week (yield 42%, based H_3_tmidc). Anal. Calcd. (%) for C_14_H_18_N_12_O_12_Zn: C, 27.49; H, 2.94; N, 27.49. Found: C, 27.68; H, 2.85; N, 27.44. IR spectra (KBr, cm^−1^): 3555 (s), 3400 (s), 3136 (s), 1536 (s), 1493 (s), 1398 (s), 1288 (s), 1167 (s), 1097 (s), 777 (m), 673 (m), 508 (m). ^1^HNMR (DMSO, 400 MHz), δ = 9.39 (s, 1H, tetrazole-CH), δ = 5.74(s, 2H, CH_2_).

#### 3.2.2. Syntheses of Complex [Fe(H_2_tmidc)_2_(H_2_O)_2_]·2H_2_O (**2**)

We added 2 mL of water solution, which contained FeSO_4_·7H_2_O (0.02 mmol), to 2 mL of methanol solution, which contained H_3_tmidc (0.02 mmol), and whisked until well combined. We transferred the mixture to a 10 mL reaction bottle, placed the bottle into a 25 mL hydrothermal reactor, and heated the reactor at 353 K for 12 h. Then we brought the oven to room temperature. Light yellowish green crystals with top-quality were harvested (yield 53%, based on H_3_tmidc). In complex **2**, if Fe ion remained in a +2 oxidation state, the theoretical analysis calculated (%) for C_14_H_18_FeN_12_O_12_ (602.25): C, 27.92; H, 3.01; N, 27.91; if Fe ion was +3 oxidation state, the theoretical analysis calculated (%) for C_14_H_17_FeN_12_O_12_ (601.25): C, 27.97; H, 2.85; N, 27.96. Experimental elemental analyses results: C, 28.19; H, 3.05; N, 28.15%. The percentage of hydrogen deviates from the experimental value. In addition, the reported Fe(III) complexes produced by light-colored ligands were very dark (such as red, dark red, brown, or black) [45,46,47,48,49,50]. We took a closer look at the color of complex **2**—light yellowish green is a good description. Green is typical of iron divalent compounds, H_3_tmidc ligand is pale yellow. Thus, Fe ion may remain in the +2 oxidation state in complex **2**. IR spectra (KBr, cm^−1^): 3431 (s), 3129 (s), 1557 (s), 1535 (s), 1489 (s), 1394 (m), 1273 (m), 1169 (m), 1109 (m), 773 (m), 670 (m), 509 (m).

### 3.3. Preparation of the Related Solutions

#### 3.3.1. Solutions Used in Protein Binding Studies

The reserve solution of BSA or HSA was Tris-HCl buffer solution (pH = 7.36, containing 0.05 mol·L^−1^ Tris, 0.2 mol·L^−1^ NaCl) of 5.00 × 10^−5^ mol·L^−1^. They were kept in a freezer at 273–277 K. The reserve solution was diluted with the same buffer to obtain the working solution. The reserve fluid of complexes **1** and **2** were a dimethylsulfoxide (DMSO) solution of 7.5 × 10^−4^ mol·L^−1^.

#### 3.3.2. Solutions Used in Cytotoxic Activity Evaluation

RPMI-1640 complete medium could be obtained by mixing RPMI-1640 medium (containing 100 U mL^−1^ penicillin, 100 μg mL^−1^ streptomycin) and FBS at a volume ratio of 9:1. 3-(4,5-dimethylthiazole-2-yl)-2,5-diphenyl tetrazolium bromide (MTT) was dissolved in a phosphate buffer saline (pH = 7.2–7.4), then we filtered the bacteria through the 0.22 m sterile microporous membrane. The filtrate was stored in a refrigerator at 277 K and the concentration of MTT was 5.0 mg mL^−1^. The working solution was diluted with RPMI-1640 complete medium.

### 3.4. Determination of Crystal Structure

We selected the crystal of the complex with perfect quality and glued it to the top of a thin glass thread. All measurements were made on a Bruker D8 Venture photon area-detector with Mo-Kα radiation at 298(2) K. The frames were integrated with the Bruker APEX3 software package. The Bruker SHELXTL software package was used to solve the structure [51]. The non-hydrogen atoms were refined anisotropically, while H atoms were refined isotropically. Water H atoms were found from the differential Fourier residual peak and were then locked there. Other H atoms were positioned geometrically and refined as riding atoms.

### 3.5. Molecular Docking Studies

The molecular docking software AutoDock 4.2 was used to predict the combination of the two metal complexes with HSA/BSA. The configurations of HSA and BSA were obtained from the Protein Data Bank (PDB IDs are 5X52 and 6QS9, respectively). Prior to docking, all of the small ligands and “chain B” in the X-ray diffraction structures were deleted, polar H atoms were added, and Gasteiger charges were added to the protein. The grid points was set at 52 × 56 × 60 for HSA and 60 × 60 × 60 for BSA with a grid spacing of 0.375. The grid center coordination was set as (28.015, 4.807, 21.804) for HSA and (−9.064, −0.642, 24.695) for BSA. The number of individuals was set at 150, the maximum number of evaluations was set to 2,500,000. Moreover, 100 hybrid GA-LS runs were computed for each docking.

## 4. Conclusions

Two new complexes [M(H_2_tmidc)_2_(H_2_O)_2_]·2H_2_O (M = Zn (**1**), Fe (**2**)) were obtained by the reaction of H_3_tmidc with ZnSO_4_·7H_2_O or FeSO_4_·7H_2_O. Metal ions in both complexes were six-coordinated and located in a distorted octahedral geometry. The interactions between complex **1** (or **2**) and BSA/HSA were investigated through the fluorescence technique. The results reveal that both complexes have fine binding propensity to BSA/HSA and present relatively large bond constants. Thus, BSA/HSA can be a suitable carrier for these complexes. The fluorescence quenching of BSA/HSA induced by both complexes is the static quenching mechanism and the interaction forces between both complexes and BSA/HSA are mainly van der Waals forces and hydrogen bonding. The binding constant values show that the binding abilities between both complexes and BSA are stronger than those between the complexes and HSA. The binding ability of complex **2** to BSA/HSA is stronger than those of complex **1**. In addition, both complexes and the free ligand H_3_tmidc exhibited cytotoxic activity against Eca-109. The IC_50_ values indicate that the antitumor activities of both complexes are superior to that of the free ligand H_3_tmidc. These findings are significant for further investigating the BSA/HSA interaction and biological activities of the coordination compounds involving imidazole and tetrazole groups.

## Figures and Tables

**Table 1:** Crystal data and structure refinement data of complexes **1** and **2**.

	1	2
Empirical formula	C_14_H_18_N_12_O_12_Zn	C_14_H_18_FeN_12_O_12_
Formula weight	611.77	602.25
Temperature, K	293(2)	298(2)
Crystal size, mm^3^	0.20 × 0.19 × 0.17	0.22 × 0.20 × 0.17
Crystal system	Monoclinic	Monoclinic
Space group	*P*2_1_/*C*	*P*2_1_/*C*
*a*, Å	9.1846(5)	9.2443(3)
*b*, Å	18.9797(12)	18.8504(7)
*c*, Å	6.9919(5)	6.9777(3)
*α*, deg	90	90
*β*, deg	111.887(2)	111.5800(10)
*γ*, deg	90	90
Volume, Å^3^	891.8(3)	1130.69(7)
*Z*	2	2
Calculated density, g cm^−3^	1.796	1.769
Absorption coefficient, mm^−1^	1.177	0.758
*F*(000), e	624	616
*θ* range for data collection, deg	3.213–27.561	3.207–27.573
Index ranges	−11 ≤ h ≤ 11	−9 ≤ h ≤ 12
	−24 ≤ k ≤ 24	−24 ≤ k ≤ 24
	−8 ≤ l ≤ 9	−9 ≤ l ≤ 9
Reflections collected/unique	15196/2599	16235/2596
*R*_int_	0.0225	0.0234
Data/restraints/parameters	2599/0/179	2596/0/178
Final indices *R*1/*wR*2 [*I* > 2*σ*(*I*)]	0.0267/0.0672	0.0288/0.0712
Final indices *R*1/*wR*2 (all data)	0.0307/0.0692	0.0339/0.0735
Goodness-of-fit on (*F*^2^)	1.056	1.073
Δ*ρ*_fin_ (max/min), e.Å^−3^	0.299/−0.377	0.383/−0.277
CCDC number	2024298	2024304

**Table 2:** Selected bond lengths (Å) and angles (^o^) for complexes **1** and **2**.

Complex 1		Complex 2	
Zn(1)-O(5)	2.1361(11)	Fe(1)-O(5)	2.1031(11)
Zn(1)-O(5)#1	2.1361(11)	Fe(1)-O(5)#1	2.1032(11)
Zn(1)-N(1)	2.0760(11)	Fe(1)-N(1)	2.1407(11)
Zn(1)-N(1)#1	2.0760(11)	Fe(1)-N(1)#1	2.1408(11)
Zn(1)-O(1)	2.1465(11)	Fe(1)-O(1)	2.1840(10)
Zn(1)-O(1)#1	2.1465(11)	Fe(1)-O(1)#1	2.1840(10)
O(5)-Zn(1)-O(5)#1	180	O(5)-Fe(1)-O(5)#1	180
N(1)-Zn(1)-O(5)	91.61(4)	O(5)-Fe(1)-N(1)	90.85(4)
N(1)-Zn(1)-O(5)#1	88.39(4)	O(5)#1-Fe(1)-N(1)	89.15(4)
N(1)#1-Zn(1)-O(5)	88.39(4)	O(5)-Fe(1)-N(1)#1	89.15(4)
N(1)#1-Zn(1)-O(5)#1	91.61(4)	O(5)#1-Fe(1)-N(1)#1	90.85(4)
N(1)-Zn(1)-N(1)#1	180	N(1)-Fe(1)-N(1)#1	180
O(5)-Zn(1)-O(1)#1	88.99(4)	O(5)-Fe(1)-O(1)#1	88.44(4)
O(5)#1-Zn(1)-O(1)#1	91.00(4)	O(5)#1-Fe(1)-O(1)#1	91.56(4)
N(1)-Zn(1)-O(1)#1	100.43(4)	N(1)-Fe(1)-O(1)#1	102.61(4)
N(1)#1-Zn(1)-O(1)#1	79.58(4)	N(1)#1-Fe(1)-O(1)#1	77.39(4)
O(5)-Zn(1)-O(1)	91.00(4)	O(5)-Fe(1)-O(1)	91.56(4)
O(5)#1-Zn(1)-O(1)	89.00(4)	O(5)#1-Fe(1)-O(1)	88.44(4)
N(1)-Zn(1)-O(1)	79.58(4)	N(1)-Fe(1)-O(1)	77.39(4)
N(1)#1-Zn(1)-O(1)	100.42(4)	N(1)#1-Fe(1)-O(1)	102.61(4)
O(1)-Zn(1)-O(1)#1	180	O(1)#1-Fe(1)-O(1)	180

Symmetry transformations used to generate equivalent atoms: #1 −*x*, −*y*, −*z*.

**Table 3:** Hydrogen bonds of complexes **1** and **2**.

D-H⋯A	*d*(D-H) (Å)	*d*(H⋯A) (Å)	*d*(D⋯A) (Å)	(D-H⋯A)(°)
Complex **1**				
O(3)-H(3)…O(2)	0.82	1.67	2.4937(16)	178.8
N(2)-H(2A)…O(6)	0.86	1.89	2.7094(19)	159.7
O(5)-H(5A)…O(4)#2	0.85	1.99	2.8061(16)	159.8
O(5)-H(5B)…O(2)#3	0.85	2.01	2.8319(16)	161.6
O(6)-H(6A)…N(6)#4	0.85	2.09	2.904(2)	160.8
O(6)-H(6B)…O(4)#5	0.85	2.14	2.977(2)	169.3
Complex **2**				
O(3)-H(3)…O(2)	0.82	1.67	2.4887(16)	179.3
N(2)-H(2A)…O(6)	0.86	1.89	2.7159(18)	159.3
O(5)-H(5A)…O(4)#2	0.85	1.95	2.7757(16)	162.0
O(5)-H(5B)…O(2)#3	0.85	1.97	2.8060(15)	168.2
O(6)-H(6A)…N(6)#4	0.85	2.09	2.910(2)	161.3
O(6)-H(6B)…O(4)#5	0.85	2.12	2.9592(19)	168.3

Symmetry transformations used to generate equivalent atoms: #2 −*x* + 1, −*y*, −*z*; #3 *x*, *y*, *z* + 1; #4 *x* + 1, −*y* + 1/2, *z* + 1/2; #5 *x*, −*y* + 1/2, *z* + 1/2.

**Table 4:** *K*_sv_, *K*_q_, *K*_b_, n values, and thermodynamic parameters for complex-BSA systems at three different temperatures.

Complex	*T*/K	*K*_sv_ (L∙mol^−1^)	*K*_q_ (L∙mol^−1^∙s^−1^)	*K*_b_/(L·mol^−1^)	n	Δ*H* (kJ·mol^−1^)	Δ*S* (J·mol^−1^·K^−1^)	Δ*G* (kJ·mol^−1^)
**1**	298	1.06 × 10^4^	1.06 × 10^12^	2.20 × 10^4^	0.99			−24.59
	308	6.69 × 10^3^	6.69 × 10^11^	4.50 × 10^3^	0.97	−98.31	−247.38	−22.12
	313	6.22 × 10^3^	6.22 × 10^11^	3.56 × 10^3^	0.95			−20.88
**2**	298	2.22 × 10^4^	2.22 × 10^12^	2.62 × 10^5^	1.24			−30.87
	308	2.06 × 10^4^	2.06 × 10^12^	5.94 × 10^4^	1.11	−108.59	−260.80	−28.26
	313	1.98 × 10^4^	1.98 × 10^12^	3.26 × 10^4^	1.05			−26.96

**Table 5:** *K*_sv_, *K*_q_, *K*_b_, n values, and thermodynamic parameters for complex-HAS systems at three different temperatures.

Complex	*T*/K	*K*_sv_ (L∙mol^−1^)	*K*_q_ (L∙mol^−1^∙s^−1^)	*K*_b_/(L·mol^−1^)	n	Δ*H* (kJ·mol^−1^)	Δ*S* (J·mol^−1^·K^−1^)	Δ*G* (kJ·mol^−1^)
**1**	298	1.04 × 10^4^	1.04 × 10^12^	3.33 × 10^3^	0.90			−20.84
	308	1.02 × 10^4^	1.02 × 10^12^	1.43 × 10^3^	0.81	−164.05	−480.56	−16.04
	313	5.57 × 10^3^	5.57 × 10^11^	9.53 × 10^1^	0.59			−11.26
**2**	298	2.78 × 10^4^	2.78 × 10^12^	4.80 × 10^4^	1.06			−27.77
	308	2.56 × 10^4^	2.56 × 10^12^	2.51 × 10^4^	0.99	−189.87	−543.95	−22.33
	313	1.74 × 10^4^	1.74 × 10^12^	7.18 × 10^2^	0.64			−19.61

## Data Availability

The data presented in this study are available on request from the corresponding author.

## Supplementary Materials

The following supplementary materials can be downloaded at: https://www.mdpi.com/article/10.3390/molecules27061886/s1, Syntheses of 2-((1*H*-tetrazol-1-yl)methylene)-1*H*-imidazole-4,5-dicarboxylic acid (H_3_tmidc). Figure S1: The three-dimensional structure of complex **2** linked by hydrogen bonds (dashed lines). Figure S2: Coordination environment of the Zn(II) ion in complex **1** with the atom numbering scheme, displacement ellipsoids are drawn at the 30% probability level. Figure S3: The two-dimensional structure of complex **1** linked by hydrogen bonds (yellow dashed lines). Figure S4: The fluorescence emission spectra of BSA in the absence and presence of H_3_tmidc, complexes **1** and **2** at 298 K. Figure S5: The Stern–Volmer curves for quenching of complex **1** with BSA at 298 K (**a**), 308 K (**b**), and 313 K (**c**). Figure S6: The Stern–Volmer curves for quenching of complex **2** with BSA at 298 K (**a**), 308 K (**b**), and 313 K (**c**). Figure S7: Double-log plots of complex **1** quenching effects on BSA fluorescence at 298 K (**a**), 308 K (**b**), and 313 K (**c**). Figure S8: Double-log plots of complex **2** quenching effects on BSA fluorescence at 298 K (**a**), 308 K (**b**), and 313 K (**c**). Figure S9: The quenching effect of complex **1** on HSA fluorescence at 298 K (**a**), 308 K (**b**), and 313 K (**c**). Arrow shows the emission intensity changes upon the increasing concentration of complex **1**. Figure S10: The quenching effect of complex **2** on HSA fluorescence at 298 K (**a**), 308 K (**b**), and 313 K (**c**). Arrow shows the emission intensity changes upon the increasing concentration of complex **2**. Figure S11: The Stern–Volmer curves for quenching of complex **1** with HSA at 298 K (**a**), 308 K (**b**), and 313 K (**c**). Figure S12: The Stern–Volmer curves for quenching of complex **2** with has at 298 K (**a**), 308 K (**b**), and 313 K (**c**). Figure S13: Double-log plots of complex **1** quenching effects on HSA fluorescence at 298 K (**a**), 308 K (**b**), and 313 K (**c**). Figure S14: Double-log plots of complex **2** quenching effects on HSA fluorescence at 298 K (**a**), 308 K (**b**), and 313 K (**c**). Figure S15: Synchronous fluorescence spectra of HSA in the presence of increasing amounts of complex **1** at Δ*λ* = 15 nm (**a**) and at Δ*λ* = 60 nm (**b**). Arrow shows the emission intensity changes upon the increasing concentration of complex **1**. Figure S16: Synchronous fluorescence spectra of HSA in the presence of increasing amounts of complex **2** at Δλ = 15 nm (**a**) and at Δ*λ* = 60 nm (**b**). Arrow shows the emission intensity changes upon the increasing concentration of complex **2**. Figure S17: ^1^H NMR spectra of complex **1**. Figure S18: ^1^H NMR spectra of complex **2**. Figure S19: IR spectra of complex **1**. Figure S20: IR spectra of complex **2**. Cif data and checkcif for complexes **1** and **2**.
