# Supplementary material for: Synthesis and Structure Elucidation of Two Essential Metal Complexes: In-Vitro Studies of Their BSA/HSA-Binding Properties, Docking Simulations, and Anticancer Activities
Source: Molecules. 2022 Mar 14;27(6):1886. doi: 10.3390/molecules27061886 (PMC8948964; doi:10.3390/molecules27061886)

checkCIF/PLATON report

Structure factors have been supplied for datablock(s) b

THIS REPORT IS FOR GUIDANCE ONLY. IF USED AS PART OF A REVIEW PROCEDURE FOR PUBLICATION, IT SHOULD NOT REPLACE THE EXPERTISE OF AN EXPERIENCED CRYSTALLOGRAPHIC REFEREE.

No syntax errors found. CIF dictionary Interpreting this report

Datablock: b

```
Bond precision:  C-C = 0.0020 A          Wavelength=0.71073

Cell:            a=9.2443(3)     b=18.8504(7)        c=6.9777(3)
                 alpha=90        beta=111.580(1)     gamma=90
Temperature:     298 K
```

	Calculated	Reported
Volume	1130.70(7)	1130.69(7)
Space group	P 21/c	P 21/c
Hall group	-P 2ybc	-P 2ybc
Moiety formula	C14 H14 Fe N12 O10, 2(H2 O)	C14 H14 Fe N12 O10, 2(H2 O)
Sum formula	C14 H18 Fe N12 O12	C14 H18 Fe N12 O12
Mr	602.25	602.25
Dx,g cm-3	1.769	1.769
Z	2	2
Mu (mm-1)	0.758	0.758
F000	616.0	616.0
F000’	617.00	
h,k,lmax	12,24,9	12,24,9
Nref	2612	2596
Tmin,Tmax	0.846,0.987	0.719,0.746
Tmin’	0.846	

```
Correction method= # Reported T Limits: Tmin=0.719 Tmax=0.746
AbsCorr = MULTI-SCAN

Data completeness= 0.994          Theta(max)= 27.573

R(reflections)= 0.0288( 2321)     wR2(reflections)= 0.0735( 2596)

S = 1.073                  Npar= 178
```

```
The following ALERTS were generated. Each ALERT has the format
       test-name_ALERT_alert-type_alert-level.
Click on the hyperlinks for more details of the test.
```

Alert level G

```
PLAT007_ALERT_5_G Number of Unrefined Donor-H Atoms .............          6 Report
PLAT232_ALERT_2_G Hirshfeld Test Diff (M-X)  Fe1      --O1       .        6.5 s.u.
PLAT232_ALERT_2_G Hirshfeld Test Diff (M-X)  Fe1      --N1       .        5.2 s.u.
PLAT794_ALERT_5_G Tentative Bond Valency for Fe1       (II)      .       2.06 Info
PLAT910_ALERT_3_G Missing # of FCF Reflection(s) Below Theta(Min).          3 Note
PLAT912_ALERT_4_G Missing # of FCF Reflections Above STh/L=  0.600         14 Note
PLAT933_ALERT_2_G Number of OMIT Records in Embedded .res File ...          1 Note
PLAT978_ALERT_2_G Number C-C Bonds with Positive Residual Density.          4 Info
PLAT992_ALERT_5_G Repd & Actual _reflns_number_gt Values Differ by          1 Check
```

```
   0 ALERT level A = Most likely a serious problem - resolve or explain
   0 ALERT level B = A potentially serious problem, consider carefully
   0 ALERT level C = Check. Ensure it is not caused by an omission or oversight
   9 ALERT level G = General information/check it is not something unexpected

   0 ALERT type 1 CIF construction/syntax error, inconsistent or missing data
   4 ALERT type 2 Indicator that the structure model may be wrong or deficient
   1 ALERT type 3 Indicator that the structure quality may be low
   1 ALERT type 4 Improvement, methodology, query or suggestion
   3 ALERT type 5 Informative message, check
```

It is advisable to attempt to resolve as many as possible of the alerts in all categories. Often the minor alerts point to easily fixed oversights, errors and omissions in your CIF or refinement strategy, so attention to these fine details can be worthwhile. In order to resolve some of the more serious problems it may be necessary to carry out additional measurements or structure refinements. However, the purpose of your study may justify the reported deviations and the more serious of these should normally be commented upon in the discussion or experimental section of a paper or in the "special_details" fields of the CIF. checkCIF was carefully designed to identify outliers and unusual parameters, but every test has its limitations and alerts that are not important in a particular case may appear. Conversely, the absence of alerts does not guarantee there are no aspects of the results needing attention. It is up to the individual to critically assess their own results and, if necessary, seek expert advice.

Publication of your CIF in IUCr journals

A basic structural check has been run on your CIF. These basic checks will be run on all CIFs submitted for publication in IUCr journals (*Acta Crystallographica*, *Journal of Applied Crystallography*, *Journal of Synchrotron Radiation*); however, if you intend to submit to *Acta Crystallographica Section C* or *E* or *IUCrData*, you should make sure that full publication checks are run on the final version of your CIF prior to submission.

Publication of your CIF in other journals

Please refer to the *Notes for Authors* of the relevant journal for any special instructions relating to CIF submission.

PLATON version of 22/12/2019; check.def file version of 13/12/2019

Datablock b - ellipsoid plot

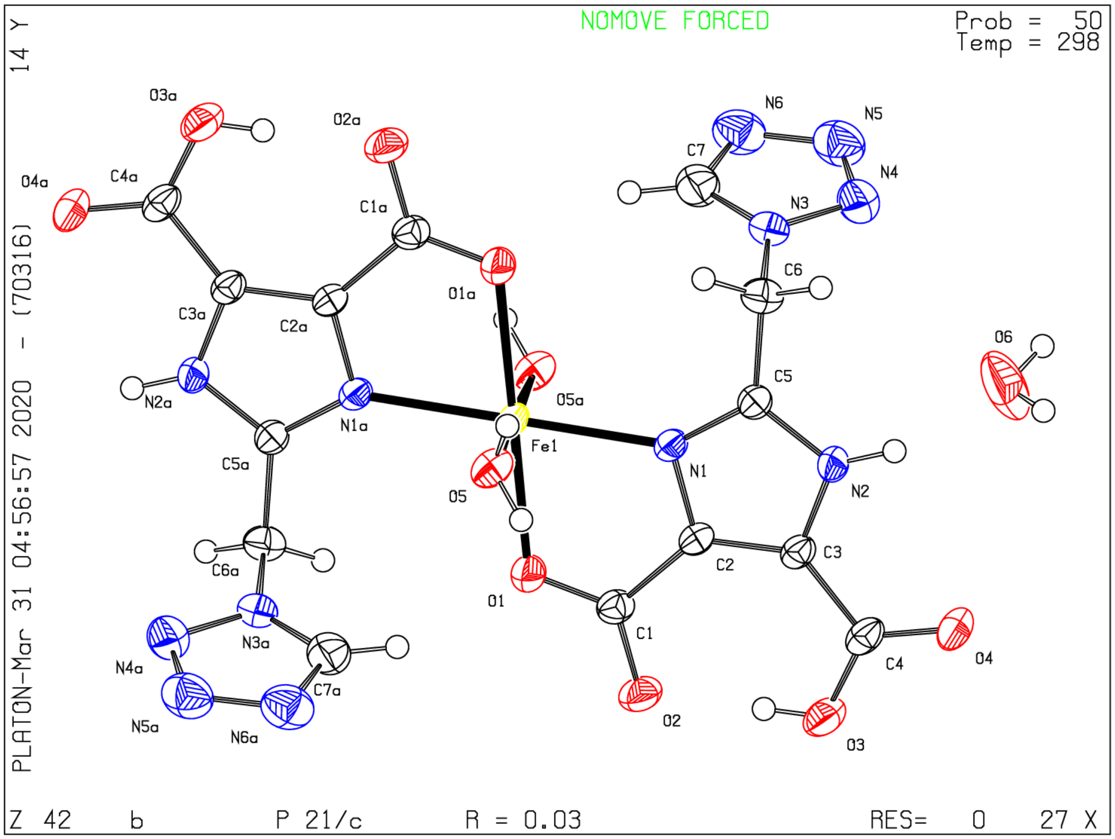

Supplement: Supplementary file 1 [file molecules-27-01886-s001.zip › molecules-1614277Supporting Information/cif-data and checkcif/2-checkcif.pdf]
