# Supplementary material for: Synthesis and Structure Elucidation of Two Essential Metal Complexes: In-Vitro Studies of Their BSA/HSA-Binding Properties, Docking Simulations, and Anticancer Activities
Source: Molecules. 2022 Mar 14;27(6):1886. doi: 10.3390/molecules27061886 (PMC8948964; doi:10.3390/molecules27061886)
Supplement: Supplementary file 1 [file molecules-27-01886-s001.zip › molecules-1614277Supporting Information/molecules-1614277 - Supplementary Materials.pdf]

molecules

Supplementary Materials

Synthesis and Structure Elucidation of Two Essential Metal Complexes: In-Vitro Studies of Their BSA/HSA-Binding Properties, Docking Simulations, and Anticancer Activities

Jun-Li Guo, Guang-Yu Liu, Rui-Ying Wang and Shu-Xiang Sun *

Syntheses of 2-((1*H*-tetrazol-1-yl)methylene)-1*H*-imidazole-4,5-dicarboxylic acid (H_3tmidc)

The ligand 2-((1*H*-tetrazol-1-yl)methylene)-1*H*-imidazole-4,5-dicarboxylic acid (H_3tmidc) was synthesized in two steps. In the first step, 2-((1*H*-tetrazol-1-yl)methylene)-1*H*-benzo[d]imidazole was synthesized according to the literature with some modifications [1]. O-phenylenediamine (80 mmol, 8.6 g) and tetranitrazoleacetic acid (88 mmol, 11.3 g) were dissolved in 100 ml 4 mol·L^{-1} hydrochloric acid. The solution was heated to 130 °C and refluxed for 10 h. After slow cooling to room temperature, the mixture was poured into iced-water. The pH value was adjusted to 8 with 40% NaOH, which gave a voluminous precipitate. The precipitate was filtered and washed with distilled water. Yield, 38.3%. In the second step, H_3tmidc was prepared according to another literature with some modifications [2]. A solution of 30% hydrogen peroxide (20 mL) was added dropwise to a solution of 2-((1*H*-tetrazol-1-yl)methylene)-1*H*-benzo[d]imidazole (13 mmol, 2.6 g) in concentrated sulfuric acid (20 mL) at 130 °C over 1.5 h. The reaction mixture was heated at 130 °C for another 10 h and then poured into ice water (ca. 80 mL). Pale yellow crystals were precipitated after standing in cold storage. The precipitation was filtered and washed with distilled water. Then yellow needle crystals were obtained by recrystallization with distilled water. Yield, 49.5%. ^{1}HNMR (DMSO, 400MHz)，δ = 9.50(s, 1H, tetrazole-CH), δ = 5.87(s, 2H, CH_2).

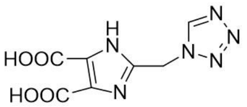

^{1}H NMR spectra

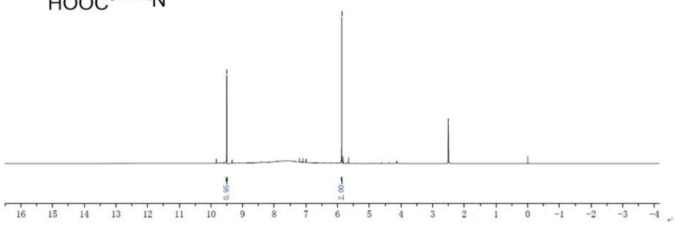

[1] Meng, X.-R.; Wu, X.-J.; Li, D.-W.; Hou, H.-W.; Fan, Y.-T. Influence of the anion on the coordination mode of an unsymmetrical N-heterocyclic ligand in Cd(II) complexes: From discrete molecule to one- and two-dimensional structures. *Polyhedron* **2010**, *29*, 2619-2628.

[2] Zheng, S.-R.; Cai, S.-L.; Pan, M.; Fan, J.; Xiao, T.-T.; Zhang, W.-G. The construction of coordination networks based on imidazole-based dicarboxylate ligand containing hydroxymethyl group. *CrystEngComm* **2011**, *13*, 883-888.

List of Figures in Supporting Information

Figure S1. The three-dimensional structure of complex **2** linked by hydrogen bonds (dashed lines).

Figure S2. Coordination environment of the Zn(II) ion in complex **1** with the atom numbering scheme, displacement ellipsoids are drawn at the 30% probability level.

Figure S3. The two-dimensional structure of complex **1** linked by hydrogen bonds (yellow dashed lines).

Figure S4. The fluorescence emission spectra of BSA in the absence and presence of H_3tmidc, complexes **1** and **2** at 298K.

Figure S5. The Stern-Volmer curves for quenching of complex **1** with BSA at 298K (a), 308K (b) and 313 K (c).

Figure S6. The Stern-Volmer curves for quenching of complex **2** with BSA at 298K (a), 308K (b) and 313 K (c).

Figure S7. Double-log plots of complex **1** quenching effects on BSA fluorescence at 298K (a), 308K (b) and 313 K (c).

Figure S8. Double-log plots of complex **2** quenching effects on BSA fluorescence at 298K (a), 308K (b) and 313 K (c).

Figure S9. The quenching effect of complex **1** on HSA fluorescence at 298K (a), 308K (b) and 313 K (c). Arrow shows the emission intensity changes upon the increasing concentration of complex **1**.

Figure S10. The quenching effect of complex **2** on HSA fluorescence at 298K (a), 308K (b) and 313 K (c). Arrow shows the emission intensity changes upon the increasing concentration of complex **2**.

Figure S11. The Stern-Volmer curves for quenching of complex **1** with HSA at 298K (a), 308K (b) and 313 K (c).

Figure S12. The Stern-Volmer curves for quenching of complex **2** with HSA at 298K (a), 308K (b) and 313 K (c).

Figure S13. Double-log plots of complex **1** quenching effects on HSA fluorescence at 298K (a), 308K (b) and 313 K (c).

Figure S14. Double-log plots of complex **2** quenching effects on HSA fluorescence at 298K (a), 308K (b) and 313 K (c).

Figue S15. Synchronous fluorescence spectra of HSA in the presence of increasing amounts of complex **1** at $\Delta\lambda$ = 15 nm (a) and at $\Delta\lambda$ = 60 nm (b). Arrow shows the emission intensity changes upon the increasing concentration of complex **1**.

Figure S16. Synchronous fluorescence spectra of HSA in the presence of increasing amounts of complex **2** at $\Delta\lambda$ = 15 nm (a) and at $\Delta\lambda$ = 60 nm (b). Arrow shows the emission intensity changes upon the increasing concentration of complex **2**.

Figure S17. ^{1}H NMR spectra of complex **1**.

Figure S18. ^{1}H NMR spectra of complex **2**.

Figure S19. IR spectra of complex **1**.

Figure S20. IR spectra of complex **2**.

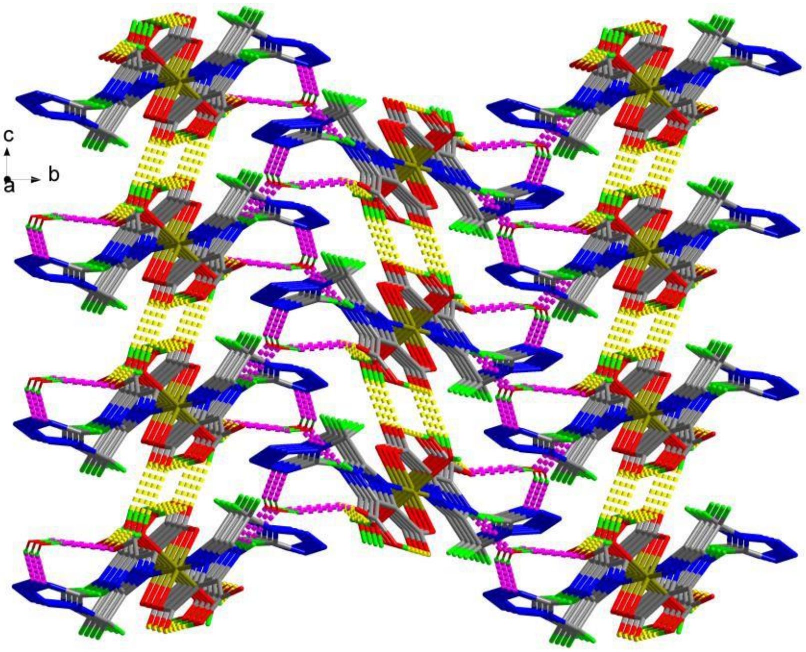

Figure S1. The three-dimensional structure of complex **2** linked by hydrogen bonds (dashed lines).

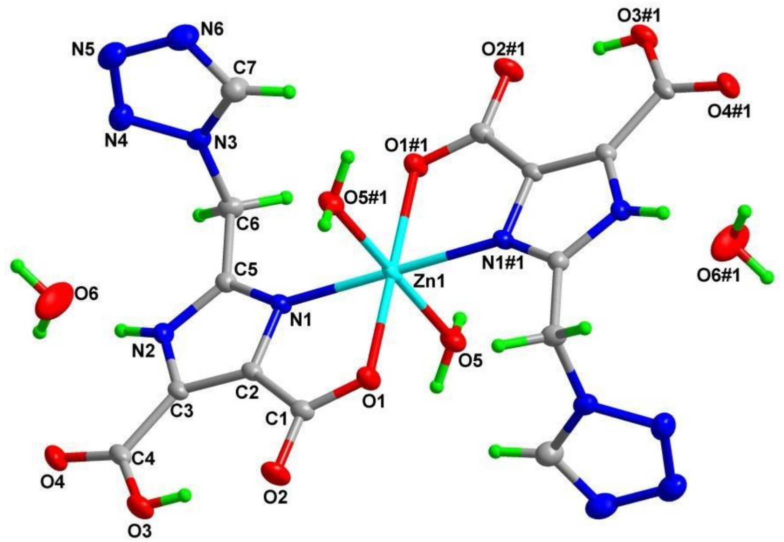

Figure S2. Coordination environment of the Zn(II) ion in complex **1** with the atom numbering scheme, displacement ellipsoids are drawn at the 30% probability level.

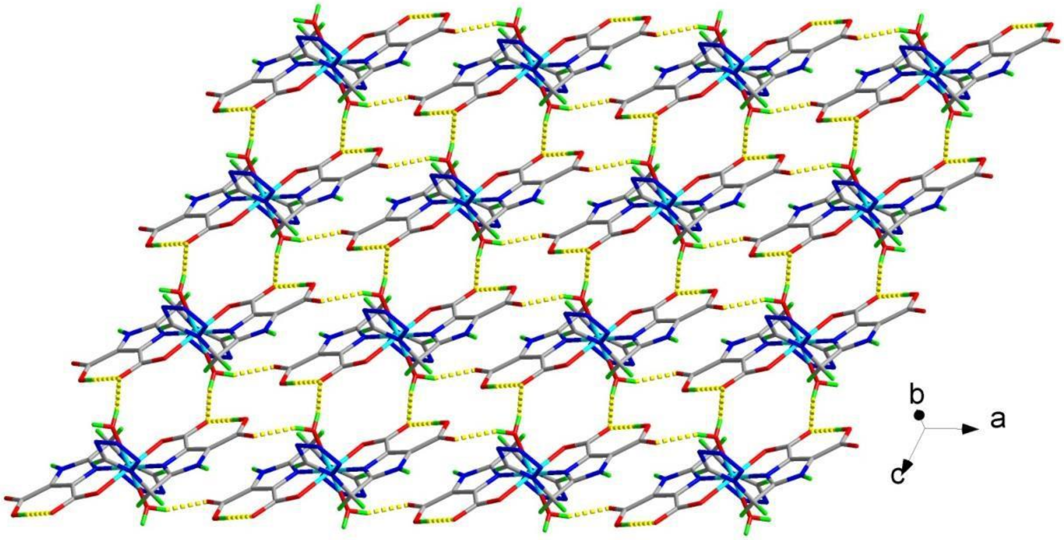

Figure S3. The two-dimensional structure of complex **1** linked by hydrogen bonds (yellow dashed lines).

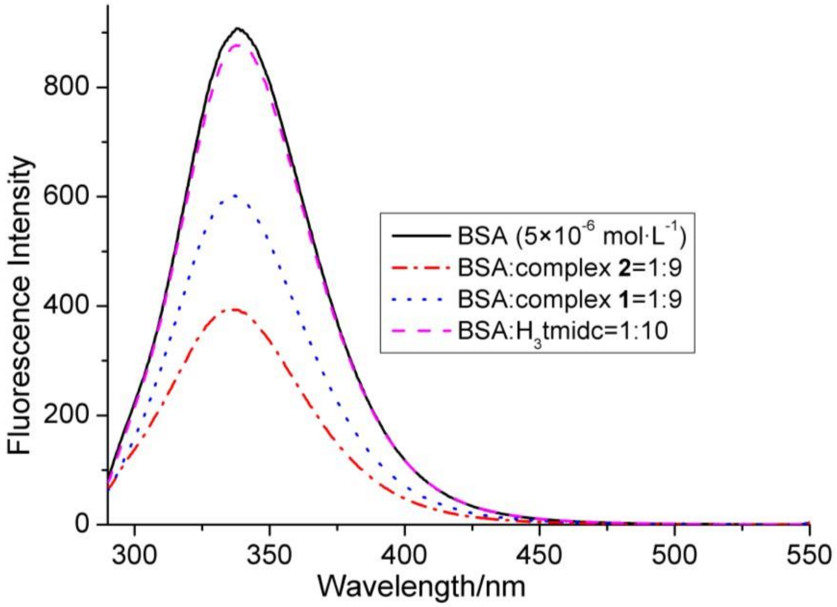

Figure S4. The fluorescence emission spectra of BSA in the absence and presence of H_3tmidc, complexes **1** and **2** at 298K.

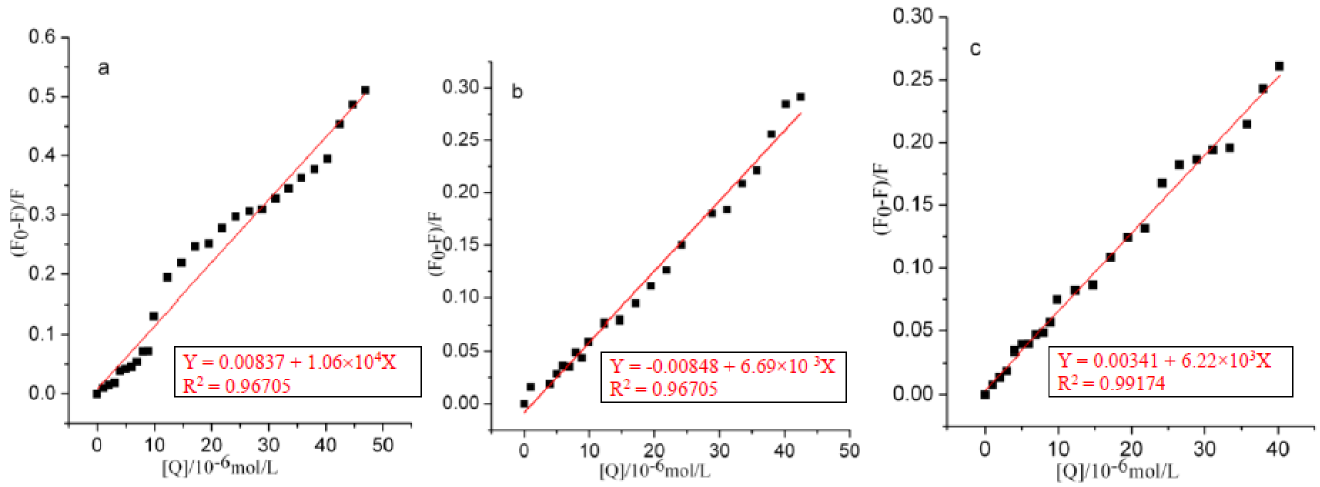

Figure S5. The Stern-Volmer curves for quenching of complex **1** with BSA at 298K (a), 308K (b) and 313 K (c).

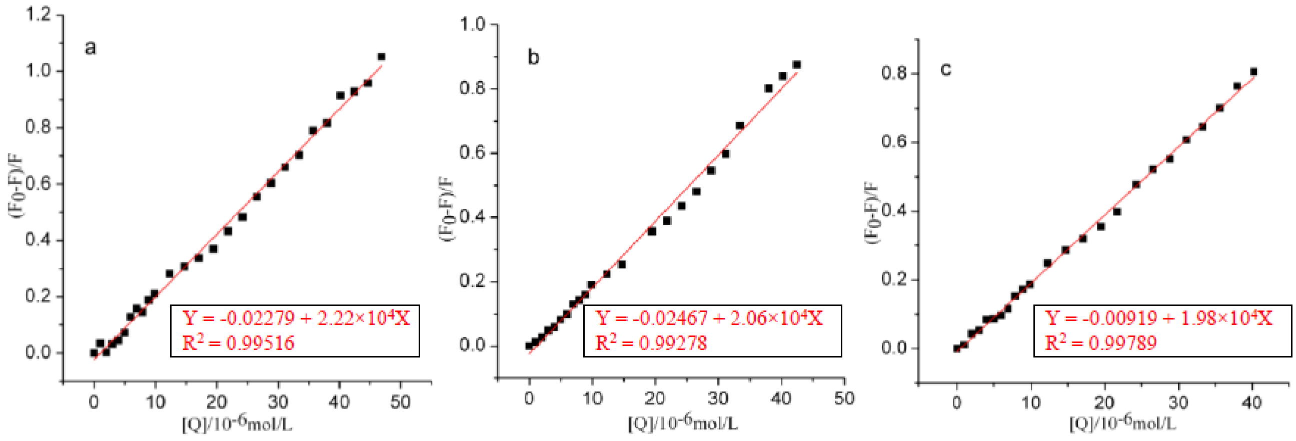

Figure S6. The Stern-Volmer curves for quenching of complex **2** with BSA at 298K (a), 308K (b) and 313 K (c).

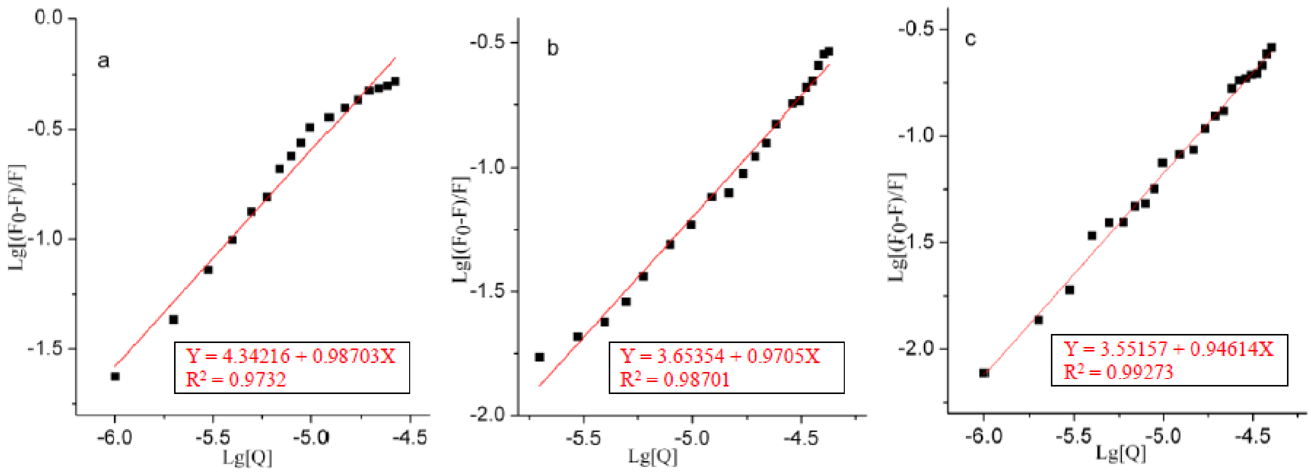

Figure S7. Double-log plots of complex **1** quenching effects on BSA fluorescence at 298K (a), 308K (b) and 313 K (c).

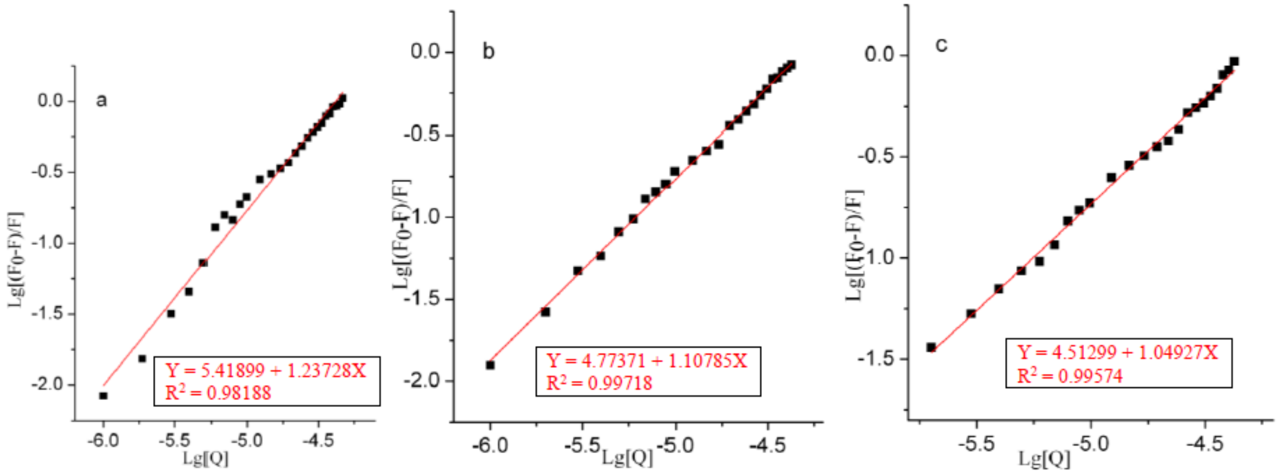

Figure S8. Double-log plots of complex **2** quenching effects on BSA fluorescence at 298K (a), 308K (b) and 313 K (c).

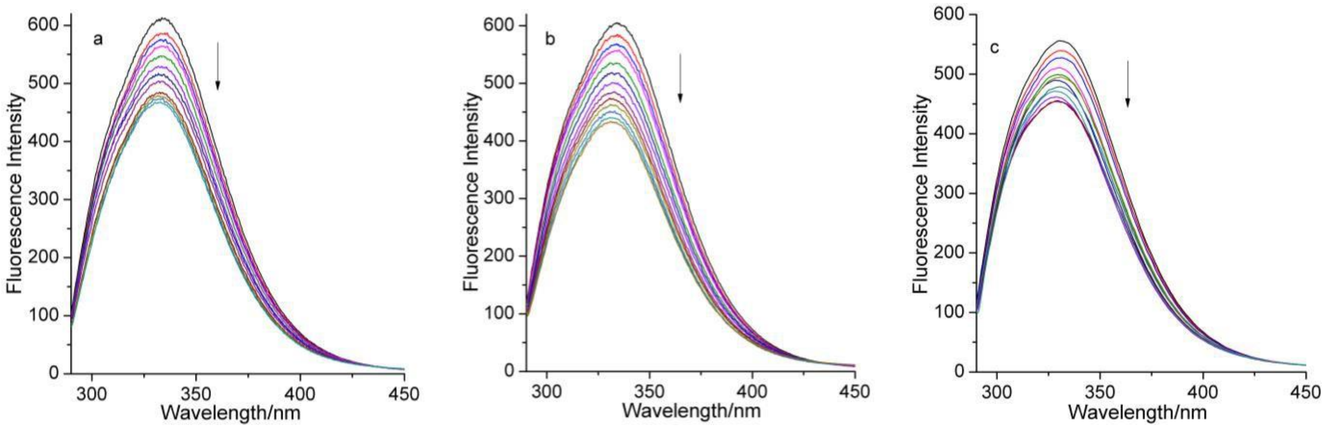

Figure S9. The quenching effect of complex **1** on HSA fluorescence at 298K (a), 308K (b) and 313 K (c). Arrow shows the emission intensity changes upon the increasing concentration of complex **1**.

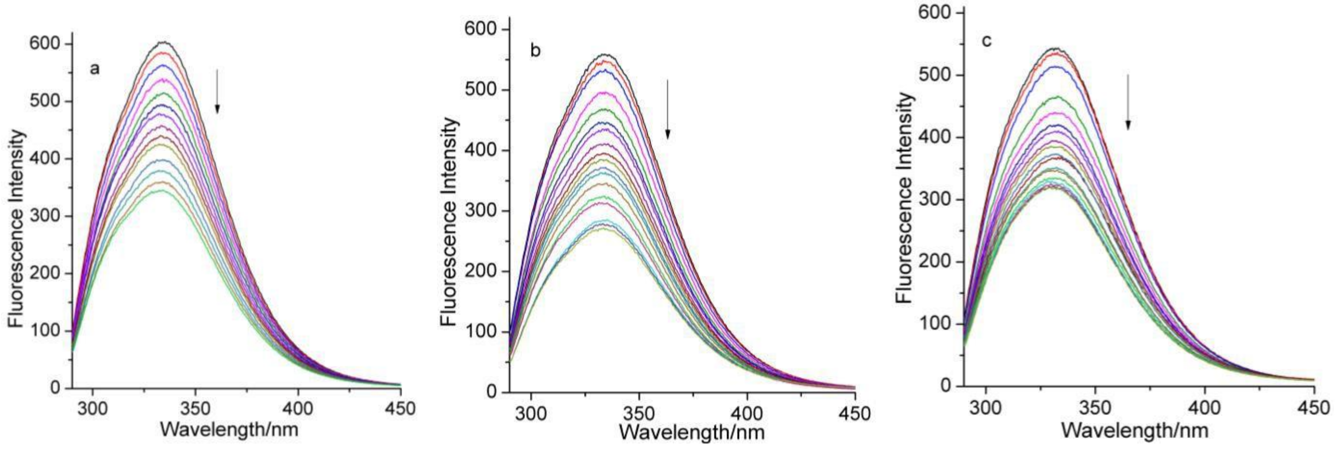

Figure S10. The quenching effect of complex **2** on HSA fluorescence at 298K (a), 308K (b) and 313 K (c). Arrow shows the emission intensity changes upon the increasing concentration of complex **2**.

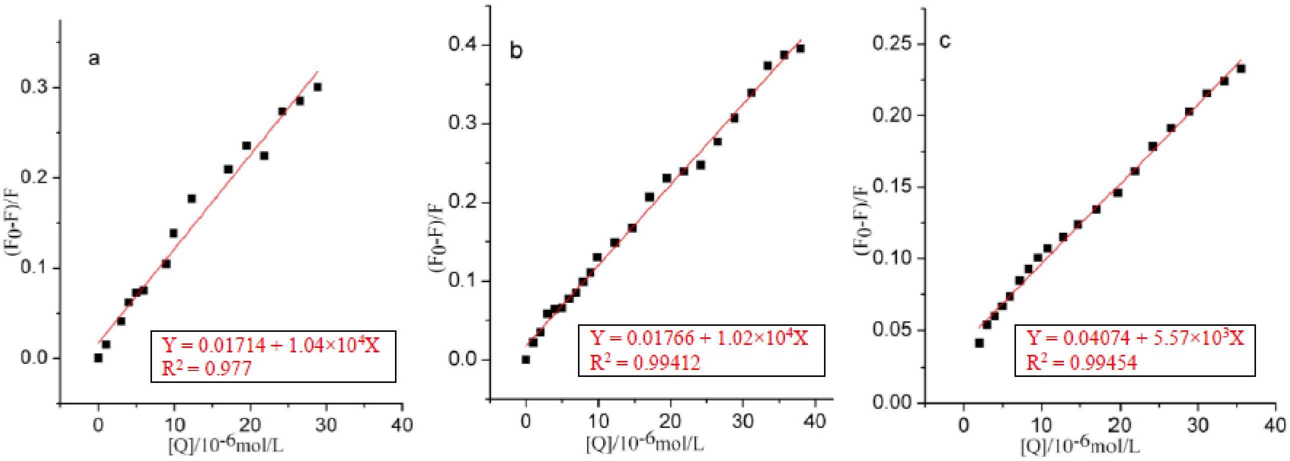

Figure S11. The Stern-Volmer curves for quenching of complex **1** with HSA at 298K (a), 308K (b) and 313 K (c).

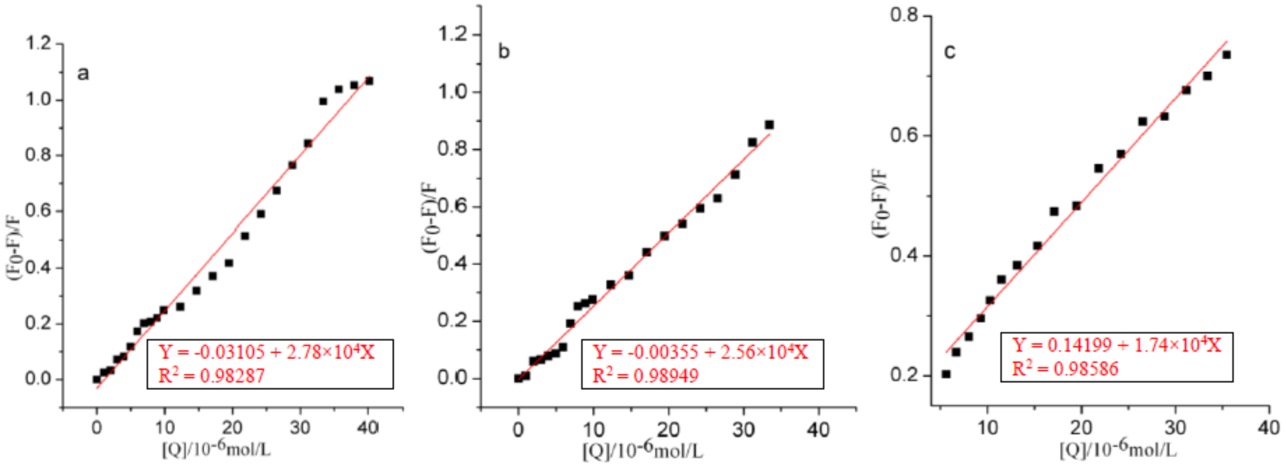

Figure S12. The Stern-Volmer curves for quenching of complex **2** with HSA at 298K (a), 308K (b) and 313 K (c).

R^2 = 0.99667

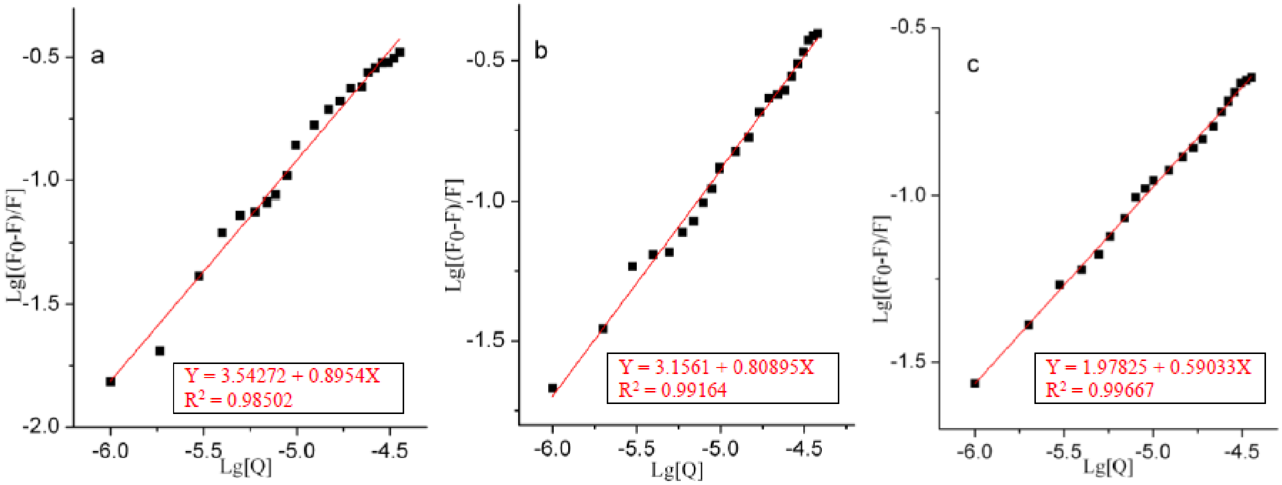

Figure S13. Double-log plots of complex **1** quenching effects on HSA fluorescence at 298K (a), 308K (b) and 313 K (c).

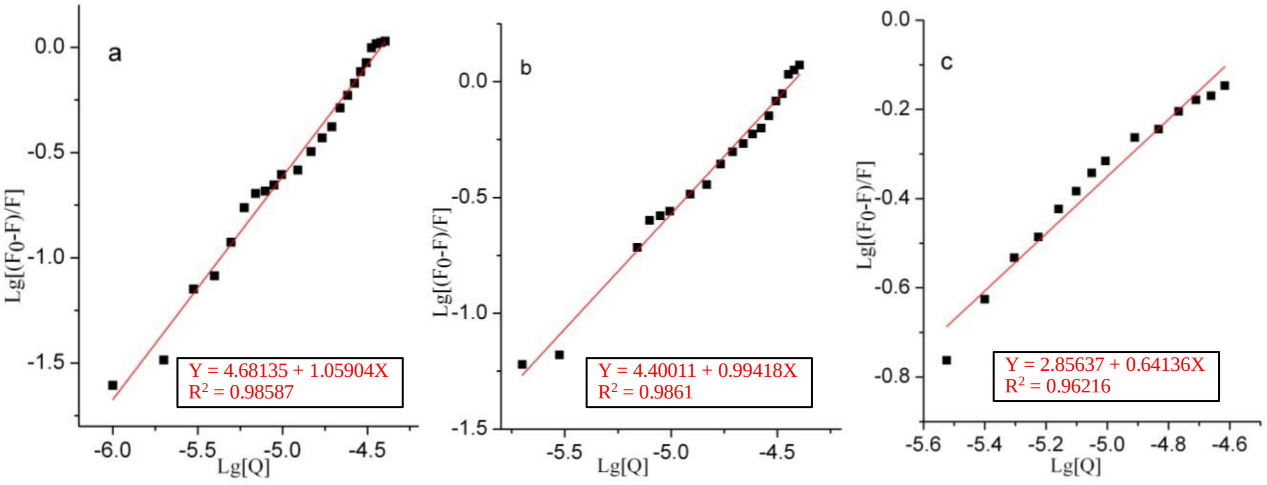

Figure S14. Double-log plots of complex **2** quenching effects on HSA fluorescence at 298K (a), 308K (b) and 313 K (c).

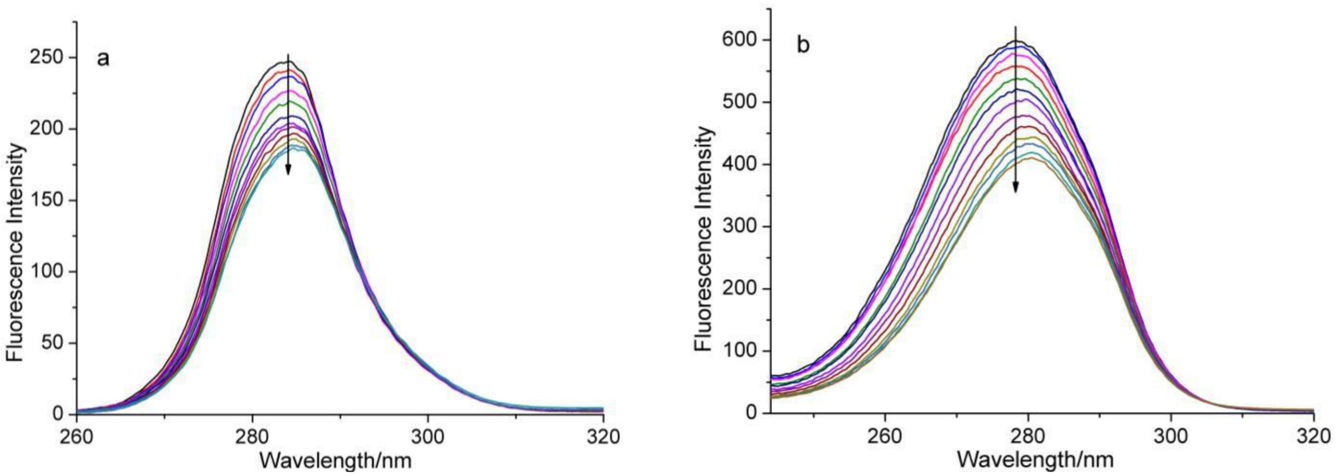

Figue S15. Synchronous fluorescence spectra of HSA in the presence of increasing amounts of complex **1** at Δλ = 15 nm (a) and at Δλ = 60 nm (b). Arrow shows the emission intensity changes upon the increasing concentration of complex **1**.

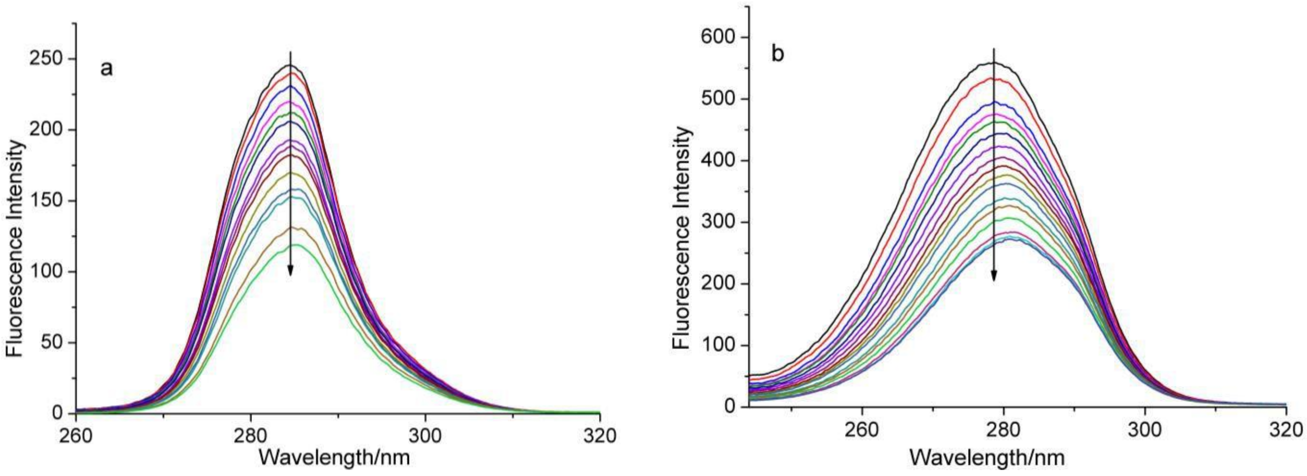

Figure S16. Synchronous fluorescence spectra of HSA in the presence of increasing amounts of complex **2** at Δλ = 15 nm (a) and at Δλ = 60 nm (b). Arrow shows the emission intensity changes upon the increasing concentration of complex **2**.

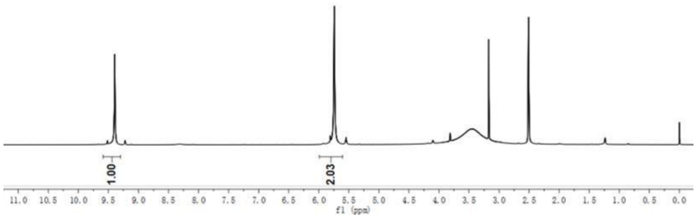

Figure S17. ^{1}H NMR spectra of complex **1**.

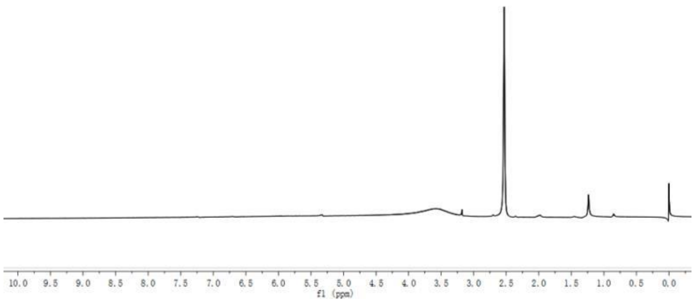

Figure S18. ^{1}H NMR spectra of complex **2**.

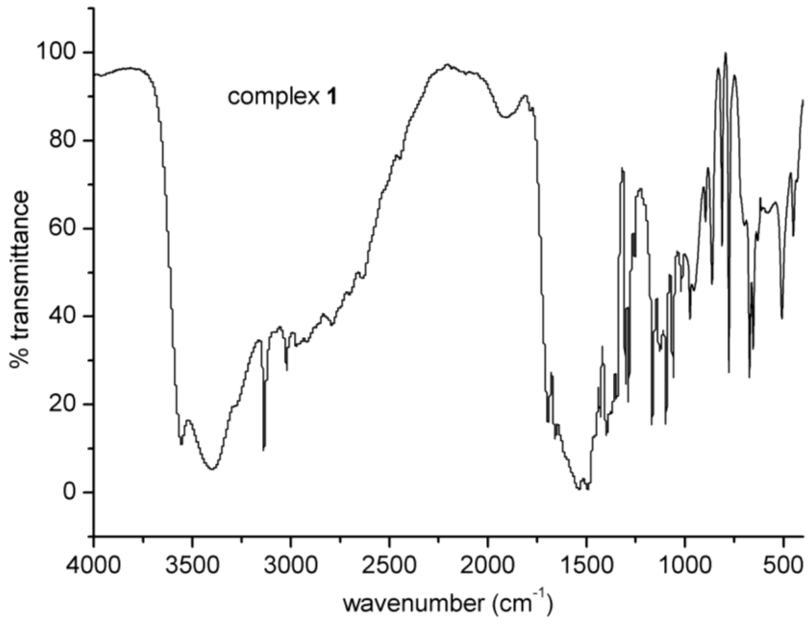

Figure S19. IR spectra of complex **1**.

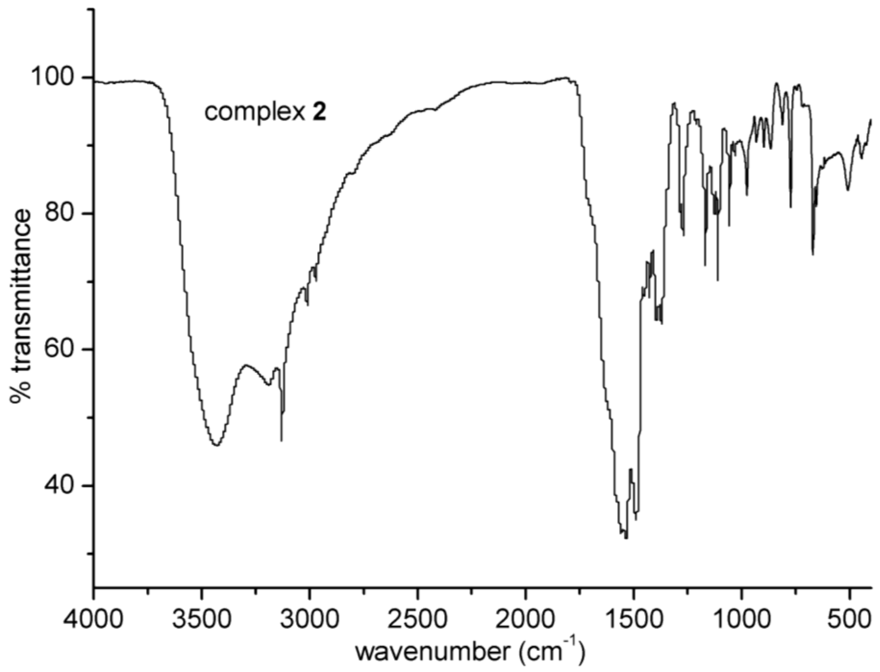

Figure S20. IR spectra of complex **2**.
